# How are sexual orientations, gender identities and expressions, and sex characteristics (SOGIESC) addressed in UN conventions, treaty bodies, and decisions: a scoping review

**DOI:** 10.1186/s12992-025-01180-x

**Published:** 2025-12-22

**Authors:** Mathieu Seppey, Yulia Bodryzlova, Muriel Mac-Seing, Gabriel Girard, Christina Zarowsky

**Affiliations:** 1 École de Santé publique, Université de Montréal, Montréal, Canada; 2 Centre de recherche en santé publique, Montréal, Canada; 3 Aix Marseille Université, Inserm, IRD, SESSTIM, ISSPAM, Marseille, France

**Keywords:** Human rights, United Nations treaties, LGBTIQ+, Inclusion, SOGIESC, Norms

## Abstract

**Background:**

Sexual orientations, gender identities and expressions, and sexual characteristics (SOGIESC) concepts are not mentioned in human rights treaties but are increasingly present in the activity of UN treaty bodies, resulting for instance in decisions, general comments and recommendations, concluding observations. Scholars have developed an increasing interest for this inclusion of SOGIESC in international law, more specifically human rights. However, the literature and case work of treaty bodies are not uniform but consist of a diversity of issues and cases under different conventions. A clearer understanding of the legal context could improve human rights advocates’ engagement and facilitate the inclusion of SOGIESC concepts in international human rights law.

**Methods:**

This scoping review aims to better understand how treaty bodies address diverse SOGIESC issues, while illustrating information gaps and contemporary debates through academic literature. A search of two UN treaty bodies’ databases resulted in the inclusion of 58 documents related to treaty bodies’ activity and a search of six academic literature databases resulted in the inclusion of 57 documents that were thematically analysed. Following these key emerging themes, gaps are identified through a “Gender and Sexual Diversity Health and Wellbeing Critical Analysis”.

**Results:**

While SOGIESC diversity is not mentioned within any treaty, it is nonetheless present through an evolutive interpretation of human rights treaties by treaty bodies. This evolutive interpretation can be represented by a chronology of legal events and key emerging and maturing themes such as privacy, freedom of expression and peaceful assembly, family life, health, education, sexism and stereotypes, non-refoulement and torture, bodily integrity and the best interest of the child.

**Discussion:**

Various disparities based on SOGIESC are now recognised through these key issues. However cis-heteronormativity is seldomly mentioned as a root cause, leaving these disparities “unexplained” and unchanged. SOGIESC concepts are also understood separately from other intersectionalities. Key UN treaty bodies still have not made the connection with SOGIESC, including for example the Committee on the Elimination of Racial Discrimination and the Committee on the Rights of Persons with Disabilities. An important gap appears in academic literature around the mobilisation and applicability of human rights treaties by/for diverse SOGIESC individuals and communities. Treaty bodies can be further mobilised through their general comments or recommendations, concluding observations, and decisions to provide further authoritative norms protecting SOGIESC rights. Specific attention should address substantiating claims properly, with improved investigating and charging practices and with stronger partnerships with SOGIESC diverse communities.

**Supplementary Information:**

The online version contains supplementary material available at 10.1186/s12992-025-01180-x.

## Introduction

People with diverse sexual orientations, gender identities and expressions, and sex characteristics (SOGIESC)^1^ suffer from various forms of abuse and discrimination (e.g., intersex genital mutilations (IGM) for infants, murder of trans* people, bullying, barriers to services, SOGIESC criminalisation) [1]. These discriminations impact a multitude of social determinants of health (e.g., income, education, support networks, social and physical environments, health practices and services) that ultimately lead these communities to experience worse health outcomes than cisgender (having a gender identity corresponding to its sex assigned at birth), heterosexual (being exclusively attracted to people of the other sex), and endosex (having a body corresponding to medical and social norms) populations. Mental, physical, and social health are each impacted (e.g., respectively: higher rates of suicide, depression; higher rates of sexual abuse and violence and sexually transmitted diseases such as HIV or hepatitis A & B; higher rates of homelessness, eating disorders)) [1].

While discrimination based on SOGIESC is often addressed domestically, it also has ramifications at the global level. The Yogyakarta Principles address these global discriminatory norms from a human rights perspective by asserting diverse SOGIESC rights, through various structural determinants of health (e.g., access to education, housing, health, and participation in cultural or political life) [2, 3]. They were first published in 2007 by the International Commission of Jurists and International Service for Human Rights. They were developed in two stages, with the first reiterating the obligations for States to ensure holistic, universal, and non-discriminatory rights and freedom for diverse SOGIESC communities, with the aim of eliminating human rights violations [2]. Acknowledging a decade of significant advancements, a second version (2017) updated these obligations with the inclusion of sex characteristics within the principles and additional human rights to protect: the right to access technologies and information, to cultural diversity, to bodily integrity, or not to be criminalised [3]. Despite being non-binding, these principles are important attempts to establish human rights norms regarding SOGIESC. They illustrate the need to address cis-heteronormativity^2^ in global governance and are now widely used by international organisations, such as the United Nations (UN), as key human rights tools [4].

Norms can be created through the language used or omitted [5], the human rights instruments adopted or rejected, and the adoption of principles by key actors [6]. An interesting facet of the UN’s work is the creation of norms through legal processes, such as the treaty bodies’ work in relation to their respective conventions. While SOGIESC issues are absent from these treaties, UN treaty bodies’ activities (e.g., decisions, concluding observations, or general comments and recommendations) affect how SOGIESC issues are addressed and dealt with, making these activities global determinants of health for diverse SOGIESC communities.

The objective of this scoping review is to better understand how these concepts (SO, GIE, SC) are included in UN treaty bodies’ activities and review the recent developments and debates of this inclusion. The review does not address impacts of discrimination based on SOGIESC but will illustrate key themes (e.g., privacy, non-refoulement, best interest of the child) at the intersections of these concepts and treaty bodies’ work. Highlighting these intersections is an important step in the process of inclusion of SOGIESC concepts, by clearly situating these concepts’ place in international human rights law (IHRL) and by identifying key gaps that can be addressed by the human right sector.

## Methods

### Design of the review

This review is part of a larger review process exploring the inclusion of SOGIESC concepts in global governance, more specifically its legal and political contexts (the later part being in review process). This present article reviews the legal context of global governance and suggests the research question: How is the intersection of SOGIESC concepts and international human rights addressed in the treaty bodies’ activities? This scoping review method was chosen due to the breadth of the question, the interdisciplinarity of the evidence, and the need to address gaps in the literature [7, 8]. The review follows the PRISMA-ScR checklist [9] and the five steps of [7]: (1) formulating a research question, (2) identifying the records, (3) selecting the documents, (4) extracting the data, and (5) reporting the synthesis.

### Identification of records and selection of documents

In response to the above-mentioned question, a search strategy was developed with a documentalist to identify key words in relation to SOGIESC concepts (e.g., “intersex”, “sexual orientation”, “gender identity”). We used that strategy in two UN treaty bodies’ databases (Juris database and UN Treaty body database [10, 11]) to identify treaty bodies’ activities relating to SOGIESC. These documents were selected as primary data.

To carry out the larger review (on legal and political contexts), another search strategy was developed to examine current debates about the inclusion of SOGIESC in UN institutions. This secondary data was used in both legal and political parts of the larger review. Key words and Medical Subject Headings terms were identified in relation to three concepts: SOGIESC concepts, human rights and political tools, and global governance. Specific search strategies were applied (01/03/2022) (and updated 13/08/2024) to six databases: Medline, Embase, Worldwide Political Science Abstracts, Sociological Abstracts, PAIS, and Web of Science (Appendix: Search strategy). Identified records were uploaded to the software Covidence where duplicates were removed.

**Fig. 1:**
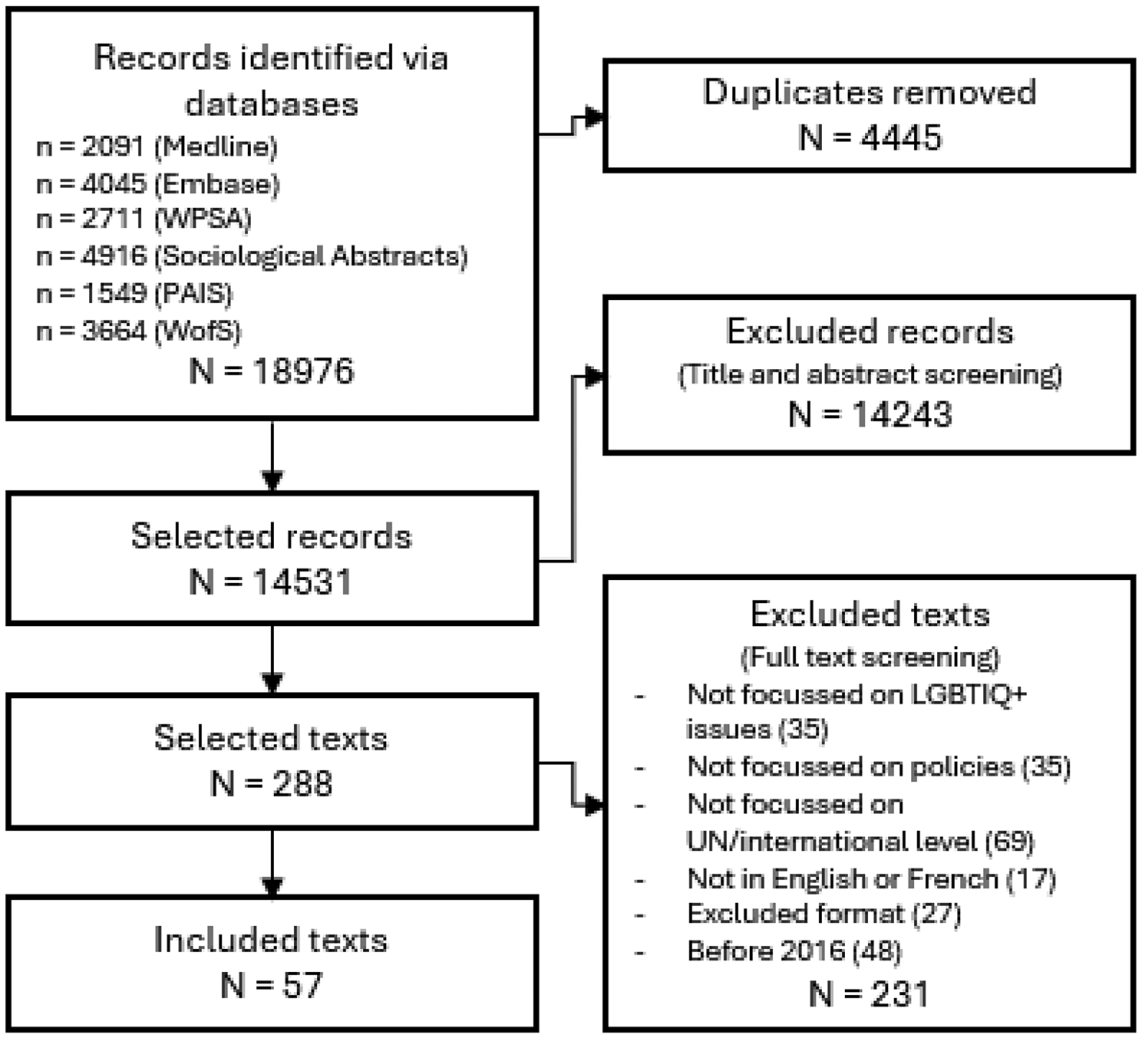
Flow Chart

A first screening, based on titles and abstract, was performed by two reviewers (MS and YB), who each screened half of the identified records with about 10% of the records screened by both reviewers. Their accordance was ensured by having discussions on mutual screened texts during the screening process. The inclusion criteria were: (1) must be about SOGIESC issues, (2) must be about human rights tools, (3) must be at the international level, and (4) must be a peer-reviewed article representing scholarly analysis (secondary sources). Following the same procedure, a second screening based on reading the full texts was subsequently performed based on the following exclusion criteria: (1) not focused on SOGIESC issues, (2) not focused on human rights tools, (3) set at a local, national, or regional level, (4) not in French or English, and (5) peer-reviewed articles published before 2016 (the year of the appointment of the Independent Expert on protection against violence and discrimination based on sexual orientation and gender identity (IE SOGI) and the year before the publication of the Yogyakarta Principles + 10). This fifth criterion was added to focus on the exploration of more contemporary debates. Following these screenings, selected documents (*N* = 57) were included in the review (Fig. 1).

### Data extraction, analysis, and synthesis

An Excel table was used to extract the data based on bibliographic (authors, years, institutions), descriptive and typological (e.g., decisions, conventions, organisations mentioned), and interpretative information (e.g., themes, barriers, facilitators, implications).

A thematic analysis [12] was conducted to identify key themes related to various international human rights treaties and to highlight gaps within the literature. This method helped structure complex and dispersed information into accessible thematic categories. The findings were synthesised through a chronology of legal events (e.g., treaties, decisions, general comments or recommendations, concluding observations) and the presentation of the emerging themes (e.g., privacy, sexism, non-refoulement, best interest of the child) present in the current literature. These results are then discussed through the application of the “Gender and Sexual Diversity Health and Wellbeing Critical Analysis”, a framework analysing the inclusion of SOGIESC in policies [1]. Gaps are identified throughout this framework’s three phases: the analysis of the disparities affecting diverse SOGIESC populations, the intersectional impacts and aspects of these disparities, and the ways to address these disparities.

## Results

**Fig. 2:**
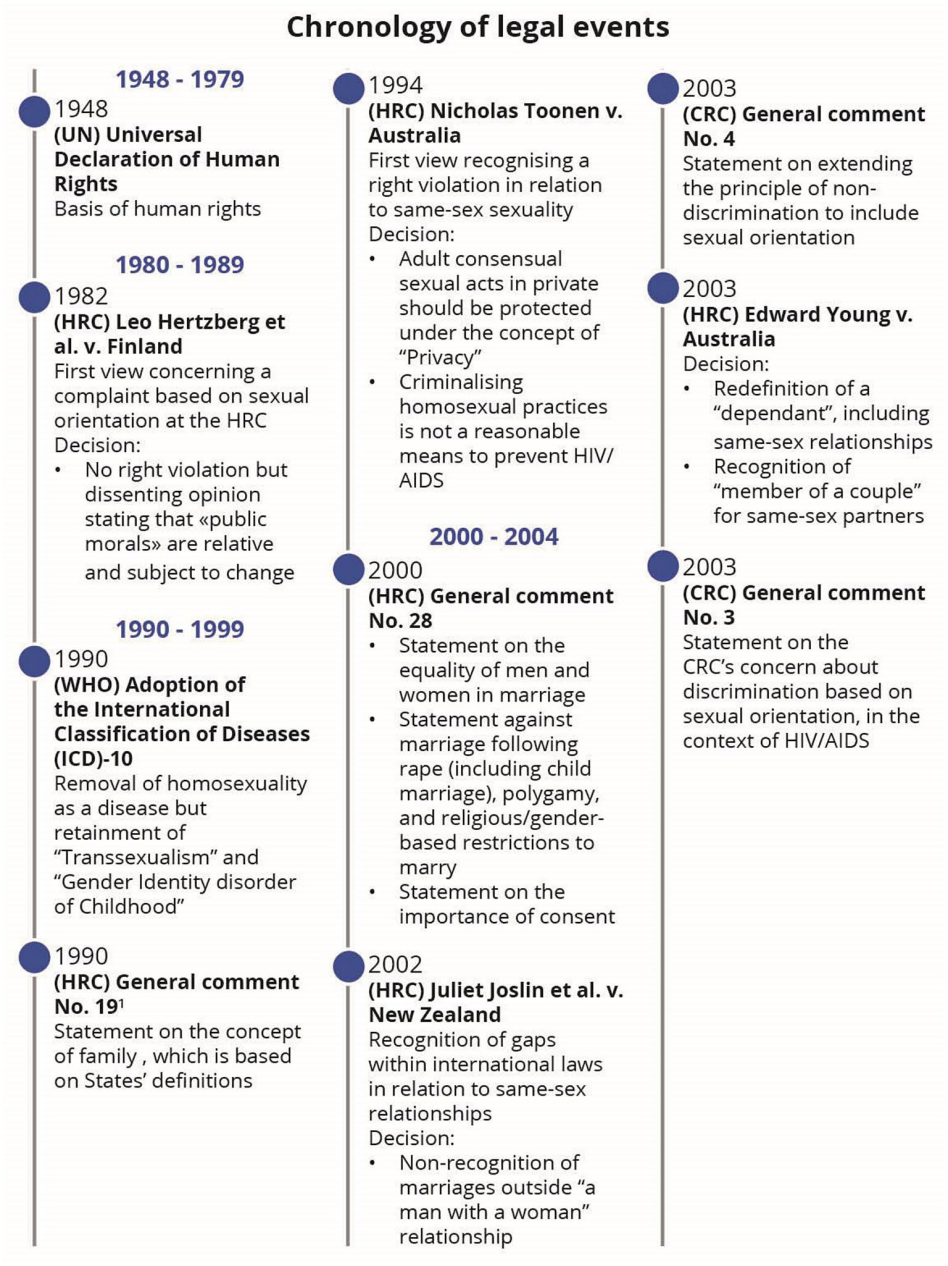

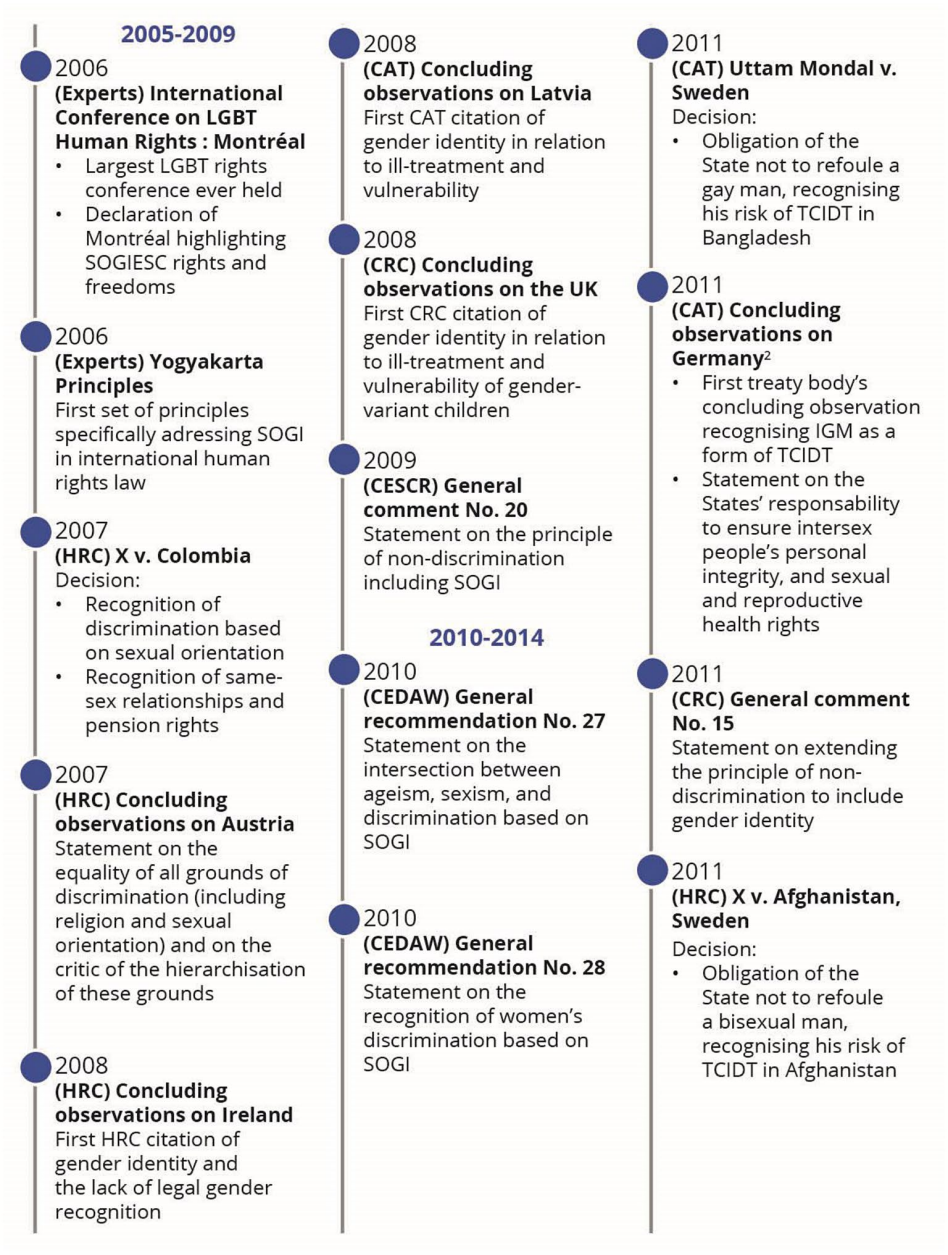

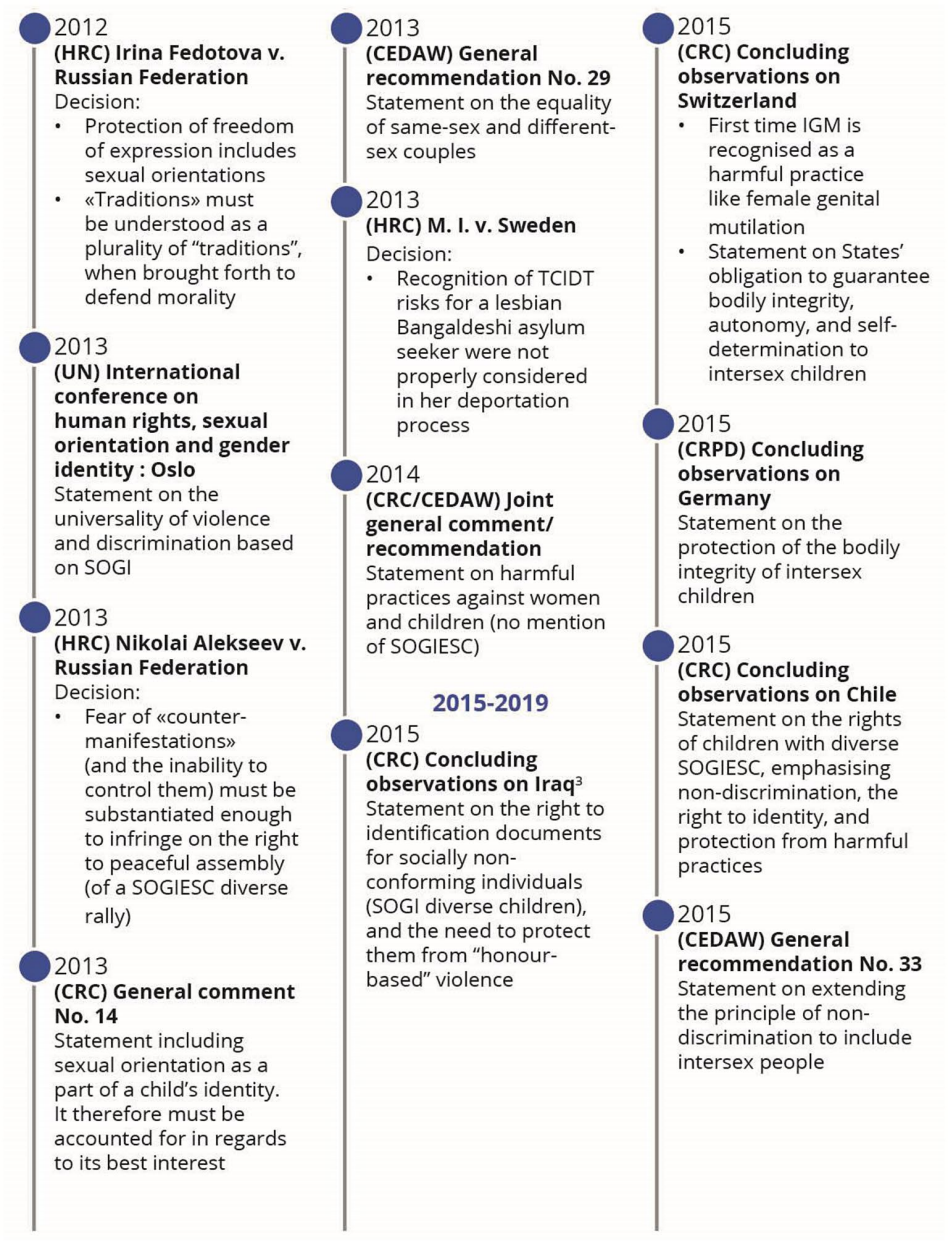

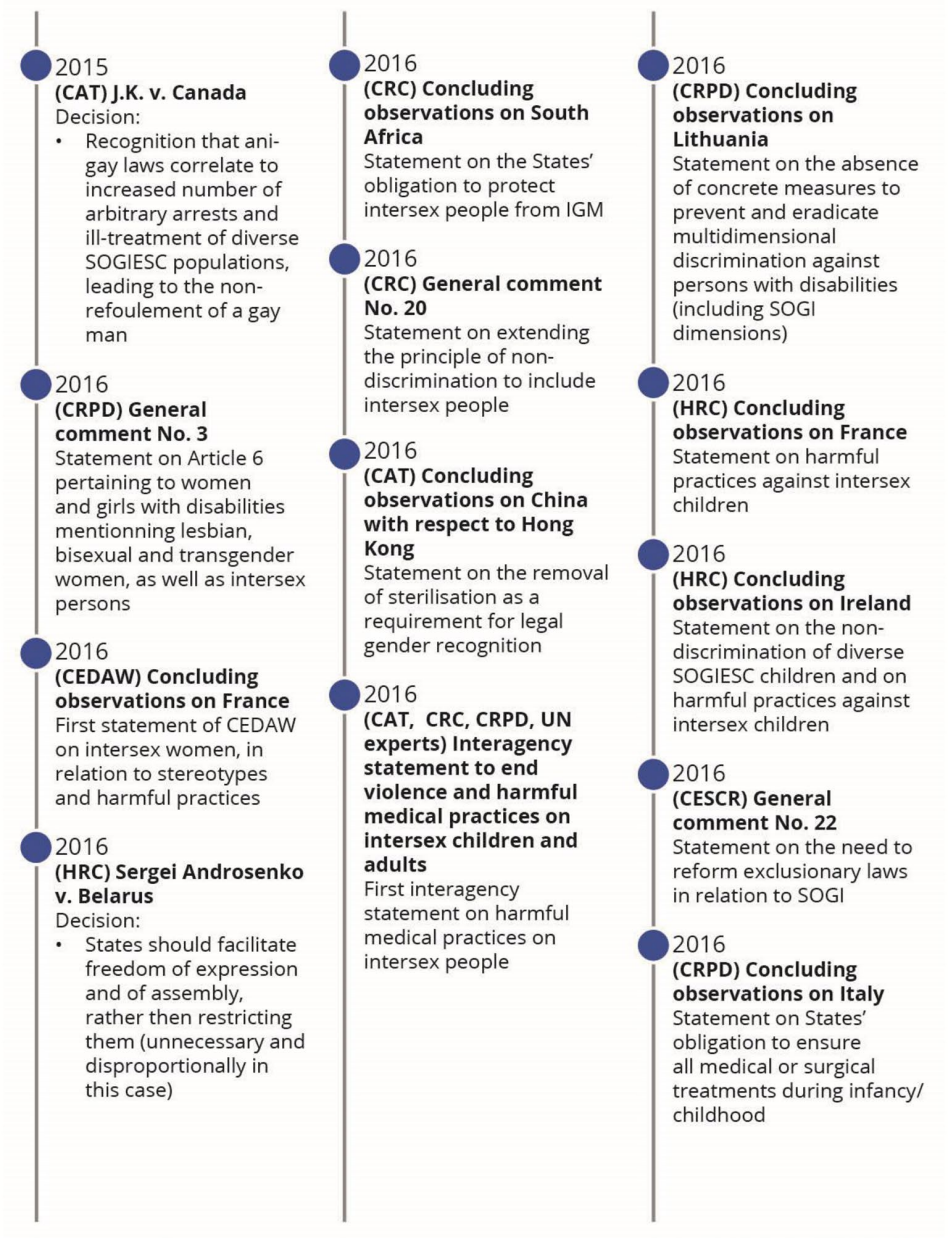

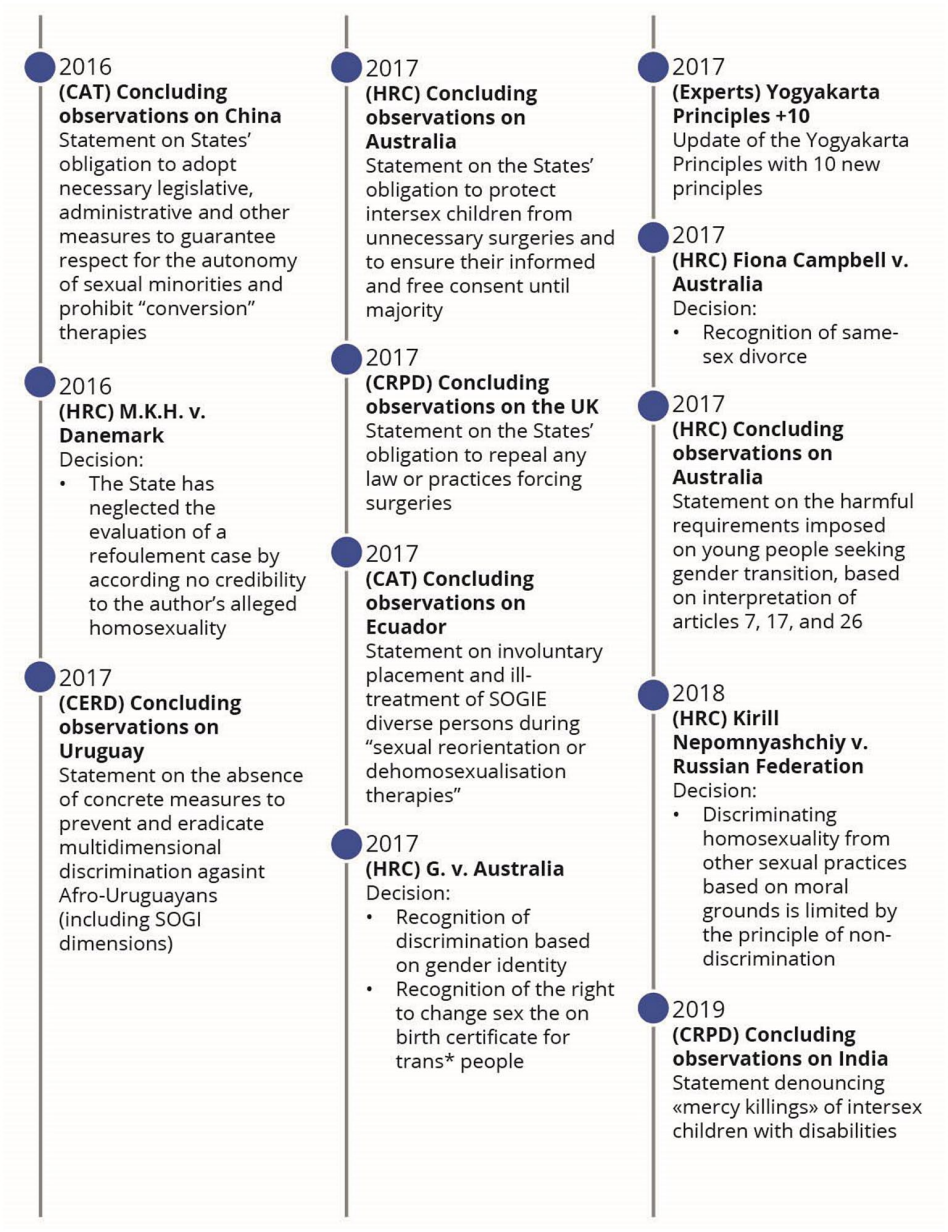

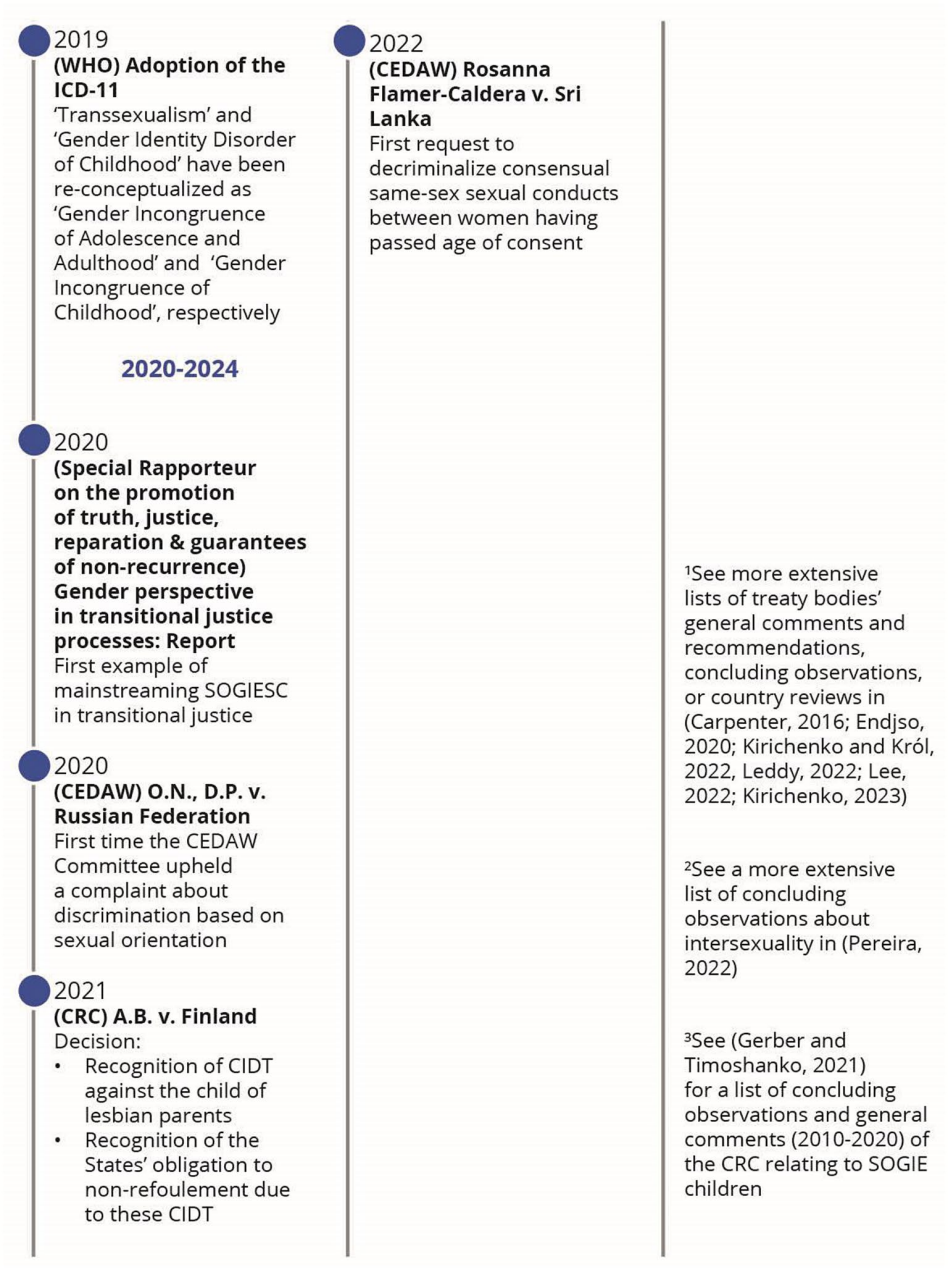
Chronology of legal events

Following the application of the search strategy, 18,976 records were identified (Fig. 1). After removing duplicates and the two screenings, 57 published articles were included in the review (Appendix: Detailed documents). From these documents, 66 events were identified as milestones in the evolution of SOGIESC human rights (Fig. 2). Results will be presented through different themes emerging from the data surrounding the different conventions and treaty bodies.

### SOGIESC within the conventions

No international human rights treaty exists specifically in relation to SOGIESC issues. While not binding nor adopted within the UN, the Yogyakarta Principles (+ 10) remains the most comprehensive interpretative tool in relation to SOGIESC human rights, and is often used by various treaty bodies, special procedures and national courts [13, 14]. They were elaborated as “mimics” of international treaties, to be understood as an authoritative international human rights tool [14]. Due to this lack of formal and specific rights, SOGIESC issues often fall at the intersection of various treaties, treaty bodies often being leaders in including SOGIESC diversity in IHRL [15, 16]. The most frequently mentioned in the included documents were: the international covenants on Civil and Political Rights (ICCPR and its Human Rights Committee (HRC)) [17] and on Economic, Social and Cultural Rights (ICESCR and its CESCR) [18], and the conventions (and committees) on the Rights of the Child (CRC) [19], and Persons with Disabilities [20], the Elimination of All Forms of Discrimination against Women (CEDAW) [21], and Racial Discrimination [22], and against Torture and Other Cruel, Inhuman or Degrading Treatment or Punishment (CAT) [23]. No mention is made about SOGIESC in any of these treaties. The application of these concepts resides within treaty bodies’ activities, such as decisions (Appendix: Legal decisions from treaty bodies) that are binding on the participating States, and interpretations made over time by treaty bodies in relation to claims made surrounding SOGIESC issues (e.g., general comments or recommendations, that are non-binding tools). All treaties contain articles on the prohibition of discrimination based on various grounds, such as “sex” or “other status”, and offer protection of rights that are comprised in diverse SOGIESC lives (see the Yogyakarta Principles for applied examples). Therefore, different conventions provide legal sites where inequities against diverse SOGIESC populations are discussed.

#### International Covenant on Civil and Political Rights (ICCPR)

Three major themes can be identified in relation to the ICCPR and its treatment of SOGIESC: privacy, freedom of expression and peaceful assembly, and marriage and its benefits. HRC’s interpretation of torture and cruel, inhuman or degrading treatment or punishment (TCIDT, and CIDT when excluding torture) will be presented in the Convention against TCIDT section, for further comparison.

##### Privacy

The HRC defines privacy as a “sphere of a person’s life in which he or she can freely express his or her identity, be it by entering into relationships with others or alone” [24] and upholds states in its protection, without arbitrary or unlawful interference (Article 17 ICCPR). In Nicholas Toonen v. Australia (1991–1994), the HRC recognised a breach in privacy intersecting with, for the first time, discrimination based on sexual orientation [25, 26]. This decision called for the repeal of a law criminalising consensual, adult, male homosexuality in private. The protection of public health (HIV epidemics) and moral grounds were not accepted as reasonable purposes for criminalising laws, which are counterproductive for public health interventions and based on a lack of consensus. This decision became a cornerstone and led to further individual complaints claiming discrimination based on sexual orientation [24] and numerous demands from the HRC to various countries, to repeal laws criminalising sexual orientation and gender identities [25].

Compared to sexual orientation, few decisions can be found about gender identity [27]. In G. v. Australia (2011–2017), Australia was again found interfering in a trans women’s privacy by refusing to change the sex on her birth certificate after her marriage [24]. This administrative refusal led to forcing disclosure of her transidentity, therefore violating her privacy. Pathologisation of trans* and intersex populations is another important barrier to legal gender recognition and could be interpreted as CIDT, under article 7 of the ICCPR [28]. Still being medicalised within the International Classification of Diseases (ICD)-11, these populations can be subjected to coercive medical treatments or diagnosis to obtain legal gender recognition (e.g., psychiatric diagnosis, sterilisation) [25, 28]. Interestingly, these coercive procedures are interpreted differently by the HRC depending on whether they are performed on cis-women (right to be free from TCIDT) or on trans* people (right to privacy), hence creating unequal jurisprudence [28]. The respect of privacy is an important basis to protect SOGIESC rights, but remain insufficient to address SOGIESC discriminations, often present at a more public level [25, 28].

##### Freedom of expression and peaceful assembly

The first HRC decision in relation to sexual orientation was with Leo Hertzberg et al. v. Finland (1979–1982), which claimed freedom of expression (article 19 [2]) was breached by the national broadcasting company censoring discussions on homosexuality [16]. The decision mentioned harmful effects on minors of broadcast discussions on homosexuality and the margin of discretion in establishing public morals; homosexuality was still considered a disease at that time. The HRC reversed its position in subsequent cases about article 19, following the general comment No. 34 stating that limiting freedom of expression cannot be based on a “single [heterosexual] tradition”. In both Irina Fedotova v. Russian Federation (2010–2012) and Kirill Nepomnyashchiy v. Russian Federation (2013–2018), limitations of a “propaganda of homosexuality among minors” was not deemed reasonable and showed the basis of discrimination (based on sexual orientation), in relation to “propaganda of heterosexuality among minors” [13].

Article 21, on the right to peaceful assembly, was brought before the HRC in 2009 through Nikolai Alekseev v. Russian Federation (2009–2013), when the authorities banned gay pride marches. States were found obligated to protect participants of peaceful assemblies, though they may be perceived as “annoying or offensive” by a majority. While not mentioned in the documents, five similar cases were filed before the HRC against Russia between 2020 and 23, underlying similar breaches to article 21 [10].

##### Marriage and benefits

Two levels of recognition exist in relation to marriage: the “institution” and the benefits. The concept of same-sex marriage was first discussed at the HRC with Juliet Joslin et al. v. New Zealand (1998–2002). Two lesbian couples were refused marriage licences due to the nonrecognition of same-sex marriage. The HRC did not recognise any breach, due to the specificity of article 23, which states both genders (“men and women” instead of spouses) [24, 29]. However, two HRC members opened the door to further cases by stating that since the benefits of marriage is a choice for heterosexual couples, the denial of those same benefits to homosexual couples, via denying marriage, could amount to discrimination based on sexual orientation. Two cases were presented in relation to pension access, Edward Young v. Australia (1999–2003) and X v. Colombia (2001–2007). Both cases demonstrated infringement of article 26 (prohibited discrimination based on sexual orientation) through the refusal of the army and State, respectively, to provide pension benefits to the remaining same-sex partners. These decisions therefore recognised the equality between unmarried hetero- and homosexual partners. In Fiona Campbell v. Australia (2012–2017), violation of article 26 was also demonstrated by the States refusing to recognise the right to divorce for a lesbian couple. While marriage benefits, such as divorce, are recognised for same-sex partners, the access to the marriage institution remains unchallenged since the unsuccessful Juliet Joslin et al. v. New Zealand case. Different arguments may contribute to a future change in the HRC’s position: (1) increasing domestic recognition of same-sex marriage, (2) the inability to demonstrate the negative impacts of same-sex marriage (e.g., deprivation, limitations to others) [30], (3) the potential degrading treatment to imply the inferiority of same-sex couples (compared to the sacredness of heterosexuality [31]), and (4) the review of “men and women” in article 23 [24]. This last argument is based on facts that the HRC members may have potentially misread the purpose of the mention of “men and women” (terms chosen to underline gender equality and not heterosexuality), and the importance of the plurality of the terms, emphasising groups over individuals [24]. Renewed interpretation may be upcoming with new discrimination claims, such as the recognition of couples with trans* individuals desiring legal gender recognition after marriage [32].

#### International Covenant on Economic, Social and Cultural Rights (ICESCR)

Only two events were found mentioning SOGIESC as listed items (general comments 20 and 22), when excluding concluding observations [33]; this illustrating unusualness of the CESCR’s addressing of SOGIESC issues outside these observations. The reviewed academic literature rarely referenced the ICESCR directly. However, two key themes emerged at the intersection of SOGIESC issues: sexual and reproductive health and education.

##### Sexual and reproductive health

The CESCR’s general comment No. 22 (on article 12) has recognised the barriers posed by exclusionary laws (e.g., criminalising homosexuality, trans-identities, and HIV non-disclosure, exposure and transmission), social stigma, and pathologisation [34]. It also recognised the States’ responsibility to protect the right to sexual and reproductive health by preventing “violence targeting LGBTI persons, … forced sterilisation … and medically unnecessary, irreversible and involuntary surgery and treatment performed on intersex infants or children” [35]; this altogether presents potential grounds for future legal advancements and advocacy [5].

##### Education

Only one peer-reviewed document discusses the intersection of the ICESCR, education, and SOGIESC, with a focus on intersexuality [36]. The document presents educational systems as highly normative and binary (male vs. female) spaces where intersex children are excluded (e.g., bullying, medical hardships) and cannot develop their human personality and sense of dignity (e.g., pedagogy based on “ab/normal” bodies).

#### Convention on the Elimination of All forms of Discrimination Against Women (CEDAW)

CEDAW was the first convention to focus on a population that is not presumed male [34]. In relation to SOGIESC, the theme of fighting sexism and stereotypes is specifically linked to this convention.

##### Sexism and stereotypes

The formulation of the articles of the convention is guided by a “transformative approach” [37], exemplified through article 5(a):

To modify the social and cultural patterns of conduct of men and women, with a view to achieving the elimination of prejudices and customary and all other practices which are based on the idea of the inferiority or the superiority of either of the sexes or on stereotyped roles for men and women [21].

While the convention was progressive, the Committee is generally prudent in addressing diverse SOGIESC intersecting with gender or further integrating SOGIESC diversity, as it is concerned about the dilution of the concept of gender in relation to ciswomen [26]. Only two cases related to SOGIESC were upheld at the CEDAW committee; two others were inadmissible due to lack of substantiation [15]. O.N., D.P. v. Russia (2017–2020), regarding a lesbian couple, was the first case to recognise States’ obligation towards clearer condemnation of discrimination against women (e.g., through legislation and legal protection) (article 2) and of social and cultural practices based on sexism and stereotypes (article 5). A second case regarding a lesbian, Rosanna Flamer-Caldera v. Sri Lanka (2018–2022), went further with the recognition of States’ obligations in eliminating discrimination against women in political and public life (article 7), ensuring equality with men before the law (article 15) and eliminating discrimination against women regarding marriage and family relations (article 16). Criminalisation of same-sex lesbian conduct was then found a breach of human rights [26]. As seen earlier with the ICCPR’s article 23 on marriage, the CEDAW does not account for identities outside the women/men binary. The inclusion of SOGIESC concepts and issues may be paradoxically difficult in this progressive convention, due to the focus on women [38].

#### Convention against Torture and Other Cruel, Inhuman or Degrading Treatment or Punishment (TCIDT) (CAT)

The CAT is increasingly used to address SOGIESC, often around the theme of non-refoulement and TCIDT.

##### Non-refoulement and TCIDT

Articles on TCIDT exist in various treaties, such as the CRC or the ICCPR. But only the CAT provides a clearer definition of the terms [35]. In its first article, torture is defined as severe physical or mental pain, aimed at punishing, intimidating, coercing or discriminating against someone, through official instigation, consent, or acquiescence [25]. CIDT is differentiated from torture in article 16, by not being as “severe”, by not necessitating each of the three components required for torture (severity of pain, purpose, intent) [25], and by being restricted to articles 10–13 regarding States’ obligations. Both the HRC and CAT committee published decisions at the intersection of SOGIESC and TCIDT, always in relation to non-refoulement cases. Non-refoulement cases often refer to diverse SOGIESC migrants being refused residency or refugee status by a country, and sent back to their country of origin. The HRC recognised violation of article 7 of the ICCPR in three cases (X v. Afghanistan, Sweden (2008–2011), M. I. v. Sweden (2012–2013), and M.K.H v. Denmark (2014–2016)). These decisions recognised the risks of TCIDT and the States’ misevaluation of the claimants’ credibility, which led to acknowledgement of the existence of the facts and the claimants’ fear of persecution. Presence of criminalising laws was found to further stigmatise diverse SOGIESC communities and maintain impunity for persecuting them [25]. These cases are important for asserting claimants’ “well-founded fear” of being persecuted [39], in relation to homo-bi-transphobic socio-cultural contexts (e.g., laws, domestic abuse). Two decisions recognised violation of article 3 by the CAT committee (Uttam Mondal v. Sweden (2007–2011) and J.K. v. Canada (2013–2015)), recognising the need to better evaluate the risks of TCIDT when extraditing someone with diverse SOGIESC. In concluding observations on different countries, the CAT committee expressed further concerns for SOGIESC communities, by recognising intersex genital mutilation [35, 40] or “sexual reorientation or dehomosexualisation therapies” [37]. Interestingly, in its first article, the CAT offers a more limiting definition of TCIDT by understanding it as: “inflicted by or at the instigation of or with the consent or acquiescence of *a public official or other person acting in an official capacity*.” [23]. By not having this limiting definition of TCIDT, the HRC can recognise broader forms of TCIDT (e.g., unofficial, private) and therefore remains a more receptive site for individual complaints [41].

#### Convention on the Rights of the Child (CRC)

While the CRC is seldomly used to address SOGIESC, the theme of the best interest of the child is emerging as an important issue, increasingly discussed.

##### Best interest of the child

The notion of the “best interest” of the child is specific to the CRC and is present in different articles. It is kept broad so it can be adapted to the different contexts and needs of every individual case. This has led the CRC committee to issue its general comment No. 14, providing a non-exhaustive, hierarchical list of elements to account in assessing a child’s best interest: the child’s view, identity, relations, protection and care, vulnerabilities, and rights to health and education [42]. That concept of “best interest” can be perceived as a tool to apply a cis-heteronormative frame on children, by guardians leaning towards “normalcy” and avoiding diverging from mainstream conceptions of that “best interest” [43]. Only one individual complaint was brought before the CRC committee in relation to sexual orientation: A.B. v. Finland (2018–2021), concerning a child with lesbian parents who were deported back to Russia [38]. The CRC committee found that the child’s best interest (article 3) was not adequately taken care of due to the lack of consideration towards the child’s view, his specific circumstances (having lesbian parents), the foreseeable abuses and bullying (linked to his parents’ sexual orientation), and the real risk of irreparable harm in relation to the refoulement.

The advancement of SOGIESC issues in the CRC can seem difficult for several reasons. First, children’s rights can be limited by the obligation for institutions and parents to protect children, since they are socially constructed in the convention as “innocent/vulnerable” and “symbol of the future” [43]. While asserted by article 12 on the right to be heard, children’s views remain limited by age and maturity and remain an evolutive concept [41, 42]. Second, the intersection of children and sexuality tends to be paradoxically over-sexualised and unwanted. It is over-sexualised through a “paedophilic gaze”, where children’s sexuality is considered in light of abuse and exploitation (articles 19 and 34) [44], and is unwanted to keep children “innocent”, as sexuality is taboo and negatively perceived [43]. This applies to all children, even more specially to diverse SOGIESC children. Third, children can be perceived as “incomplete/to be developed”, in conformity to society (articles 6, 8 and 29) [43]. Their development then becomes the opportunity for adults to create a legacy, which can be tainted by cis-heteronormativity. Again, the oversexualisation of these children, compared to cis-hetero children, reduces their capacity to form their own identities and views, which should be protected by the CRC [44]. Binary genders are homogenising children’s realities, where girls can experience genital mutilation or sexual exploitation, and boys, child conscription or school dropout [43]. On a positive note, the CRC committee has been more active in the defence of the rights of intersex children due to the need to uphold their right to bodily integrity [40, 45]. It recognises intersex genital mutilation and forms of sexual orientation change efforts as harmful practices in concluding observations on Switzerland [35], South Africa, Chile, France, and Ireland [27].

#### International Convention on the Elimination of All Forms of Racial Discrimination (CERD) and Convention on the Rights of Persons with Disabilities (CRPD)

Both CERD and CRPD have no legal case relating to diverse SOGIESC at the intersection of racism and ableism [15]. Reviewed documents only mentioned the potential of the CERD to create a space to address non-western SOGIESC diversity, and the CRPD’s importance in addressing the issues of non-discrimination and protection of bodily integrity (e.g., intersex cases) [15, 46].

## Discussion

This review of treaty bodies’ activities enables us to identify different rights and freedoms that are being tested and discussed in relation to SOGIESC concepts through various conventions and a chronology of various key legal events (e.g., treaty bodies’ decisions, general recommendations or comments). The “Gender and Sexual Diversity Health and Wellbeing Critical Analysis” [1] will be used to structure the discussion of these results, as a way to identify key gaps in relation to the inclusion of SOGIESC concepts in IHRL. This analytical tool is divided in three phases representing the inclusion process: the recognition of disparities, the comprehension of intersectional aspects, and transformative practices facilitating greater inclusion.

### Phase 1: Recognition of disparities based on SOGIESC

Various disparities based on SOGIESC are recognised through legal decisions, general comments, recommendations, and concluding observations made by treaty bodies (e.g., privacy, marriage, harmful practices, sexist issues). While recognition of these disparities is emerging, reference to their root causes is rare and is an important gap in the literature. Cis-heteronormativity or homo-bi-transphobia are rarely addressed as a cause for SOGIESC discriminations at the international level. There are a few exceptions, which are the Irina Fedotova or Kirill Nepomnyashchiy cases, where homophobia is implicitly discussed in the discreditation of “homosexual propaganda” through its comparison to “heterosexual propaganda” [13]. Even with these exceptions, disparities based on SOGIESC are often framed through cis-heteronormative assumptions that reinforce cis-heteronormative dominant models (e.g., the benefits of marriage, the importance of blood relationships, the preponderance of the biological sex over gender, or the amalgam between these concepts) [32, 47, 48]. Key issues pertaining to queer identities (and cis-heterosexual identities too) are therefore eclipsed from human rights debates, namely the question of sexual pleasures and freedoms, or the overarching pathologisation and criminalisation of identities and bodies outside the sex and gender binaries (male/female and men/women) [25, 47, 49]. As with women’s rights, SOGIESC rights struggle to be represented outside the subject of reproduction (e.g., marriage, “normal” upbringing of children) or discrimination; discriminations limited within sex and gender binaries (e.g., bringing women or SOGIESC diversity to men or cis-heterosexual standards) [26]. Being associated with these tropes of non-reproductive sexuality [50] or nonconforming binaries [26], diverse SOGIESC realities can more easily be excluded or unaccounted for in international human rights.

### Phase 2: Comprehension of the specific intersectional aspects

While SOGIESC has been recognised in relation to several topics, its inclusion in the interpretation of international human rights treaties has been incremental. Few intersections of SOGIESC diversity are explored, leaving the comprehension of these concepts to be often monolithic. Similarly to another review’s findings, complaints reviewed by treaty bodies were mostly addressing Global North countries (e.g., Australia, Sweden); more specifically to non-refoulement cases, only Global South countries were identified as the country of origin and gay and bisexual men were more clearly represented (being respectively half and a fifth of the cases) than women and transpeople (intersex people are still absent from any decision) [51]. These examples offer other representations of dominant and problematic models that are limiting the comprehension of SOGIESC around male and western experiences. Again, this correlates with the advancements of women’s rights, where representations of “new” discriminations based on gender helped to create the CEDAW and to challenge sexist practices, but failed to comprehend women as people with diverse SOGIESC and transform dominant comprehensions of SOGIESC (e.g., gender binary, duality of sexual orientations [homosexuality vs. heterosexuality]) [49, 52]. While cis-heteronormativity clearly limits the comprehension of SOGIESC, other oppressive systems (e.g., racism and colonialism [53], sexism [54]) also needs to be clearly recognised and transformed to be truly inclusive. Further exploration of the various underrepresented intersections related to SOGIESC is essential to fully understand the needs of diverse SOGIESC populations within the framework of IHRL. Emerging debates within the scientific community helps us identify some of these intersections between SOGIESC and age (e.g., bullying and abuse of diverse SOGIESC children [35, 41–44, 55, 56]), migration and conflict environments (e.g., abuses across borders, targeted communities [39, 50]), or participation in human rights (e.g., decision making by/for SOGIESC diversity [15, 57–60]). Other important intersections also deserve attention, including those between SOGIESC and racism, ableism, and diverse cultural backgrounds.

### Phase 3: Transformation of practices

By applying the “Gender and Sexual Diversity Health and Wellbeing Critical Analysis” [1] to IHRL, we can observe that while “means” of inclusion are partially present in IHRL (e.g., representations and comprehensions of SOGIESC diversity), the participation of specific “agents” of inclusion (e.g., diverse SOGIESC communities) remains problematic, which limits the presence of transformative practices. These communities’ participation is mainly performed through the mechanism of individual complaints that are highly complex [61]. Various cases have been found inadmissible, incompatible, or lacking substantiation, leading to the non-recognition of the claims [10, 51]. Preparation and resources are important to avoid these pitfalls whether by providing improved legal advice (some cases do not have representation from a legal advisor) [46] or by providing tools such as those concerning the CEDAW [62] or SOGIESC issues [51].

These procedural barriers are also reinforced by the prerequisite that international legal recourse is contingent upon the prior exhaustion of domestic remedies [61]. As IHRL lacks enforcement mechanisms [63] and is often non-binding (e.g., being general comments and recommendations and dependent on States’ cooperation to enact legal decisions), “law-abiding” States may respond positively to IHRL, while growing concerns are highlighted about its “westernisation” [13, 16, 38, 64, 65] and about counteractions such as the anti-gender movement [66]. It then falls upon domestic and international human rights advocates to pressure States to uphold human rights [16, 35, 67, 68]. Addressing domestic forms of discrimination, including criminalisation, pathologisation, and social stigmatisation, is therefore essential to unlocking the potential of international human rights mechanisms [25]. This process is already ongoing through global dialogues between national and international jurists [69] and is framed as a way to further democracy, rather than the global justice system; that argument needs to be highlighted in the advocacy for human rights nationally and internationally [70].

Finally, this review’s findings show a need for further knowledge mobilisation in relation to the intersection between SOGIESC and IHRL. This knowledge mobilisation process [71] must account for co-creation of knowledge based on SOGIESC diversity experiences and address various publics, such as the broader community, human rights agents (at both international and national levels), and diverse SOGIESC communities. Knowledge mobilisation tools must be created to nurture a “re-imagination” of IHRL, to be a truer inclusive system going beyond dualistic representations of SOGIESC (e.g., male/female, men/women, hetero/homosexuals) [72]. Knowledge mobilisation around SOGIESC in IHRL has the potential to transcend various problematics (e.g., identifying disparities between the lived experienced of diverse SOGIESC and cis-heterosexual and endosex communities, providing comprehensible tools addressing specific intersections) by offering a space for diverse SOGIESC communities’ participation and recognition. This empowering process could then be used: (1) instrumentally (by providing knowledge guiding specific actions, such as treaty bodies’ decisions), (2) conceptually (by refining understanding of specific intersecting issues), (3) strategically (by offering clearer arguments to position oneself, such as in the publication of general comments or recommendations) [71, 73], or (4) reflexively (by transforming someone’s practices and biases towards SOGIESC) [74]. The Leiden University’s Summer School on Sexual Orientation and Gender Identity in International Law (along with its associated tool, the Leiden Overview on SOGIESC in International Law [75]) serves as a strong example of a knowledge mobilisation initiative that raises awareness and trains law students on SOGIESC issues. It illustrates the broader need to engage with these issues and to mobilise and co-develop new legal knowledge in this evolving field.

### Limitations

This scoping review aimed to identify and examine SOGIESC issues in treaty bodies’ activities. Since there are various reviews on concluding observations [15, 31, 45, 46, 55], we limited this review to treaty bodies’ decisions and general comments/recommendations while mentioning concluding observations milestones to better situate the chronology. This review excluded peer-reviewed publications earlier than 2016, to concentrate on more contemporary debates taking place after the creation of the IE SOGI and the Yogyakarta Principles + 10. A short analysis of the title, abstract, and key words of the documents predating these events showed a greater focus on SO, while including GIE. The more recent documents reviewed present less focus on SO and the emergence of SC in the literature. A full retrospective could be done in the future to further understand the chronology of SOGIESC inclusion.

## Conclusion

This review of treaty bodies’ activities has traced the evolving inclusion of SOGIESC concepts within IHRL, highlighting their uneven interpretation and fragmented integration across key legal events (e.g., treaty bodies’ decisions, general comments and recommendations). While progress has been made, SOGIESC representation remains constrained by cis-heteronormative paradigms and a narrow legal operationalisation, often centred on specific comprehensions (e.g., gay men, the Global North). Key aspects of being part of the SOGIESC diversity remain unfolded (e.g., sexual pleasure, pathologisation). In addition, key intersections between SOGIESC and other axes of discrimination, such as racism, sexism, or ableism, also remain insufficiently explored, representing a clear opportunity for research and human rights. This limited and fragmented understanding of SOGIESC realities hinders the implementation of transformative practices and a more equitable, effective, and genuine inclusion of SOGIESC within IHRL. However, transformative practices can be identified to reach that goal, such as facilitating and strengthening the access to legal mechanisms, fostering democratic justice systems nationally and internationally, and co-creating and mobilising knowledge in an intersectional and participatory effort to “re-imagine what is IHRL” (in Dianne Otto’s words), beyond cis-heteronormativity.

## Supplementary Information

Below is the link to the electronic supplementary material.

Supplementary Material 1

## Data Availability

Data available in the appendices “Detailed documents” and “Legal decisions from treaty bodies”.

## Footnotes

1 We use the term “diverse SOGIESC” people, communities, or populations to describe individuals whose sexual orientation, gender identity and expression, and sex characteristics differ from heterosexual, cisgender, and endosex norms. We prefer this terminology over acronyms like LGBTIQ + and their variants, as such acronyms are often rooted in Western cultural frameworks and may carry more politically charged connotations.

2 Cis-heteronormativity refers to the concepts of “cisgenderism” and “heterosexuality” being upheld as un/conscious normality.

## Abbreviations

CAT: Convention/Committee against Torture
CEDAW: Convention/Committee on the Elimination of all forms of Discrimination Against Women
CESCR: Committee on Economic, Social and Cultural Rights
CIDT: Cruel, inhuman or degrading treatment or punishment
CRC: Convention/Committee on the Rights of the Child
HRC: Human Rights Committee
ICCPR: International Covenants on Civil and Political Rights
ICD: International Classification of Diseases
ICESCR: International Covenants on Economic, Social and Cultural Rights
IE SOGI: Independent Expert on sexual orientation and gender identity
IGM: Intersex genital mutilation
IHRL: International human rights law
LGBTIQ+: Lesbian, Gay, Bisexual, Trans*, Intersex, Queer and others
OHCHR: Office of the United Nations High Commissioner for Human Rights
SOGIESC: Sexual Orientations, Gender Identities and Expressions, and Sexual Characteristics
TCIDT: Torture and other Cruel, Inhuman or Degrading Treatment or Punishment
UNHRC: United Nations Human Rights Council
UNHCR: United Nations Refugee Agency
