# Supplementary material for: How are sexual orientations, gender identities and expressions, and sex characteristics (SOGIESC) addressed in UN conventions, treaty bodies, and decisions: a scoping review
Source: Global Health. 2025 Dec 22;22:11. doi: 10.1186/s12992-025-01180-x (PMC12836782; doi:10.1186/s12992-025-01180-x)
Supplement: Supplementary file 1 — Supplementary Material 1 [file 12992_2025_1180_MOESM1_ESM.docx]

# Appendices

## Appendix: PRISMA-ScR treaties

| **SECTION** | **ITEM** | **PRISMA-ScR CHECKLIST ITEM** | **REPORTED ON PAGE #** |
| --- | --- | --- | --- |
| **TITLE** | | | |
| Title | 1 | Identify the report as a scoping review. | Title page |
| **ABSTRACT** | | | |
| Structured summary | 2 | Provide a structured summary that includes (as applicable): background, objectives, eligibility criteria, sources of evidence, charting methods, results, and conclusions that relate to the review questions and objectives. | Abstract |
| **INTRODUCTION** | | | |
| Rationale | 3 | Describe the rationale for the review in the context of what is already known. Explain why the review questions/objectives lend themselves to a scoping review approach. | 6-7 |
| Objectives | 4 | Provide an explicit statement of the questions and objectives being addressed with reference to their key elements (e.g., population or participants, concepts, and context) or other relevant key elements used to conceptualise the review questions and/or objectives. | 7 |
| **METHODS** | | | |
| Protocol and registration | 5 | Indicate whether a review protocol exists; state if and where it can be accessed (e.g., a Web address); and if available, provide registration information, including the registration number. | Thesis protocol (not accessible) |
| Eligibility criteria | 6 | Specify characteristics of the sources of evidence used as eligibility criteria (e.g., years considered, language, and publication status), and provide a rationale. | 7-8 |
| Information sources* | 7 | Describe all information sources in the search (e.g., databases with dates of coverage and contact with authors to identify additional sources), as well as the date the most recent search was executed. | 7 |
| Search | 8 | Present the full electronic search strategy for at least 1 database, including any limits used, such that it could be repeated. | Appendix: Search strategy |
| Selection of sources of evidence† | 9 | State the process for selecting sources of evidence (i.e., screening and eligibility) included in the scoping review. | 7-8 |
| Data charting process‡ | 10 | Describe the methods of charting data from the included sources of evidence (e.g., calibrated forms or forms that have been tested by the team before their use, and whether data charting was done independently or in duplicate) and any processes for obtaining and confirming data from investigators. | 9 |
| Data items | 11 | List and define all variables for which data were sought and any assumptions and simplifications made. | 9 |
| Critical appraisal of individual sources of evidence§ | 12 | If done, provide a rationale for conducting a critical appraisal of included sources of evidence; describe the methods used and how this information was used in any data synthesis (if appropriate). | N/A |
| Synthesis of results | 13 | Describe the methods of handling and summarising the data that were charted. | 9 |
| **RESULTS** | | | |
| Selection of sources of evidence | 14 | Give numbers of sources of evidence screened, assessed for eligibility, and included in the review, with reasons for exclusions at each stage, ideally using a flow diagram. | 9-10 |
| Characteristics of sources of evidence | 15 | For each source of evidence, present characteristics for which data were charted and provide the citations. | Appendix: Detailed documents, and References |
| Critical appraisal within sources of evidence | 16 | If done, present data on critical appraisal of included sources of evidence (see item 12). | N/A |
| Results of individual sources of evidence | 17 | For each included source of evidence, present the relevant data that were charted that relate to the review questions and objectives. | Appendix: Detailed documents |
| Synthesis of results | 18 | Summarise and/or present the charting results as they relate to the review questions and objectives. | 11-31 |
| **DISCUSSION** | | | |
| Summary of evidence | 19 | Summarise the main results (including an overview of concepts, themes, and types of evidence available), link to the review questions and objectives, and consider the relevance to key groups. | 31-34 |
| Limitations | 20 | Discuss the limitations of the scoping review process. | 35 |
| Conclusions | 21 | Provide a general interpretation of the results with respect to the review questions and objectives, as well as potential implications and/or next steps. | 35-36 |
| **FUNDING** | | | |
| Funding | 22 | Describe sources of funding for the included sources of evidence, as well as sources of funding for the scoping review. Describe the role of the funders of the scoping review. | Declarations |

From: Tricco AC, Lillie E, Zarin W, O'Brien KK, Colquhoun H, Levac D, et al. PRISMA Extension for Scoping Reviews (PRISMAScR): Checklist and Explanation. Ann Intern Med. 2018;169:467–473. doi: 10.7326/M18-0850.

## Appendix: Search strategy

Medline:

((LGBT* or Lesbian* or WSW or "Women having sex with women" or Gay or Gays or MSM or "Men having sex with men" or Homosexual* or Same-sex* or Bisexual* or MSMW or "Men having sex with men and women" or WSWM or "Women having sex with women and men" or Trans or Transgender* or Transsexual* or Transidentit* or Intersex* or Queer* or Genderqueer* or Non-binar* or "Gender fluid*" or "Sexual minorit" or "Gender minorit*" or SOGI* or "Sexual orientation*" or "Gender Identit*" or "Gender expression*" or "Sexual characteristic*").ab,kf,kw,ti. or exp "Sexual and Gender Minorities"/ or exp bisexuality/ or exp femininity/ or exp gender identity/ or exp gender role/ or exp homosexuality, female/ or exp homosexuality, male/ or exp homosexuality/ or exp masculinity/ or exp sex characteristics/ or exp sex/ or exp transsexualism/ or sexuality/) AND (((Convention* or Court or Courts or Law or Laws or Legislat* or Norm* or Policy or Policies or Recommendation* or Resolution* or Right* or Treaties or Treaty) Adj7 (America* or Carribbean* or Europe* or Africa* or Asia* or Arab* or Oceania* or "United Nations" or UN or "U.N." or Global* or International or Regional or Commission* or Council* )).ab,kf,kw,ti. or ((exp human rights/ or exp jurisprudence/ or exp legislation as topic/ or exp policy making/ or exp policy/ or exp Social Control Policies/ or exp Social Norms/) and ("africa south of the sahara"/ or "commonwealth of independent states"/ or "organisation for economic cooperation and development"/ or "scandinavian and nordic countries"/ or africa, central/ or africa, eastern/ or africa, northern/ or africa, southern/ or africa, western/ or africa/ or african union/ or americas/ or asia, central/ or asia, northern/ or asia, southeastern/ or asia, western/ or asia/ or australasia/ or caribbean region/ or central america/ or europe, eastern/ or europe/ or european union/ or far east/ or international agencies/ or international cooperation/ or international health regulations/ or internationality/ or latin america/ or mediterranean islands/ or mediterranean region/ or middle east/ or north america/ or oceania/ or pacific islands/ or pan american health organization/ or south america/ or transcaucasia/ or unesco/ or united nations/ or world health organization/)))

Embase:

((LGBT* or Lesbian* or WSW or "Women having sex with women" or Gay or Gays or MSM or "Men having sex with men" or Homosexual* or Same-sex* or Bisexual* or MSMW or "Men having sex with men and women" or WSWM or "Women having sex with women and men" or Trans or Transgender* or Transsexual* or Transidentit* or Intersex* or Queer* or Genderqueer* or Non-binar* or "Gender fluid*" or "Sexual minorit" or "Gender minorit*" or SOGI* or "Sexual orientation*" or "Gender Identit*" or "Gender expression*" or "Sexual characteristic*").ab,kf,kw,ti. or exp "sexual and gender minority"/ or exp asexuality/ or exp bisexuality/ or exp femininity/ or exp gender identity/ or exp homosexuality/ or exp intersex/ or exp masculinity/ or exp sex role/ or exp sexual characteristics/ or exp transsexualism/ or exp transsexuality/ or sexual orientation/) and (((Convention* or Court or Courts or Law or Laws or Legislat* or Norm* or Policy or Policies or Recommendation* or Resolution* or Right* or Treaties or Treaty) Adj7 (America* or Carribbean* or Europe* or Africa* or Asia* or Arab* or Oceania* or "United Nations" or UN or "U.N." or Global* or International or Regional or Commission* or Council*)).ab,kf,kw,ti. or ((exp human rights/ or exp legal aspect/ or exp policy/ or exp politics/ or exp social norm/) and ("africa south of the sahara"/ or "south and central america"/ or africa/ or asia/ or balkan peninsula/ or baltic states/ or benelux/ or caribbean/ or central america/ or central asia/ or eastern europe/ or europe/ or exp "organisation for economic cooperation and development"/ or exp african union/ or exp european union/ or exp international law/ or exp united nations/ or exp world trade organization/ or far east/ or intergovernmental organization/ or international cooperation/ or international organization/ or Middle East/ or north africa/ or northern asia/ or Pacific islands/ or scandinavia/ or south america/ or south asia/ or southeast asia/ or southern europe/ or western asia/ or western europe/ )))

WPSA:

(Ti,ab,su(LGBT* or Lesbian* or WSW or "Women having sex with women" or Gay or Gays or MSM or "Men having sex with men" or Homosexual* or Same-sex* or Bisexual* or MSMW or "Men having sex with men and women" or WSWM or "Women having sex with women and men" or Trans or Transgender* or Transsexual* or Transidentit* or Intersex* or Queer* or Genderqueer* or Non-binar* or "Gender fluid*" or "Sexual minorit" or "Gender minorit*" or SOGI* or "Sexual orientation*" or "Gender Identit*" or "Gender expression*" or "Sexual characteristic*" ) or MAINSUBJECT.EXACT("Intersexuality") OR MAINSUBJECT.EXACT("Sexuality") OR MAINSUBJECT.EXACT.EXPLODE("Bisexuality") OR MAINSUBJECT.EXACT.EXPLODE("Femininity") OR MAINSUBJECT.EXACT.EXPLODE("Homosexual Relationships") OR MAINSUBJECT.EXACT.EXPLODE("Homosexuality") OR MAINSUBJECT.EXACT.EXPLODE("Lesbianism") OR MAINSUBJECT.EXACT.EXPLODE("Masculinity") OR MAINSUBJECT.EXACT.EXPLODE("Sex Differences") OR MAINSUBJECT.EXACT.EXPLODE("Sex Role Attitudes") OR MAINSUBJECT.EXACT.EXPLODE("Sex Roles") OR MAINSUBJECT.EXACT.EXPLODE("Sexual Minorities") OR MAINSUBJECT.EXACT.EXPLODE("Transsexuality")) AND (((MAINSUBJECT.EXACT("Courts") OR MAINSUBJECT.EXACT("Law") OR MAINSUBJECT.EXACT("Policy") OR MAINSUBJECT.EXACT("Rights") OR MAINSUBJECT.EXACT("Treaties") OR MAINSUBJECT.EXACT("Human Rights") OR MAINSUBJECT.EXACT("International Court") OR MAINSUBJECT.EXACT("International Law") OR MAINSUBJECT.EXACT("Norms") ) and ( MAINSUBJECT.EXACT("Intergovernmental Relations") OR MAINSUBJECT.EXACT("International Economic Organizations") OR MAINSUBJECT.EXACT("International Organizations") OR MAINSUBJECT.EXACT.EXPLODE("Commonwealth of Independent States") OR MAINSUBJECT.EXACT.EXPLODE("Cultural Groups") OR MAINSUBJECT.EXACT.EXPLODE("International Cooperation") OR MAINSUBJECT.EXACT.EXPLODE("International Courts") OR MAINSUBJECT.EXACT.EXPLODE("International Law") OR MAINSUBJECT.EXACT.EXPLODE("Internationalism") )) or ( noft(Convention* or Court or Courts or Law or Laws or Legislat* or Norm* or Policy or Policies or Recommendation* or Resolution* or Right* or Treaties or Treaty ) near/7 noft(America* or Carribbean* or Europe* or Africa* or Asia* or Arab* or Oceania* or "United Nations" or UN or "U.N." or Global* or International or Regional or Commission* or Council*) ))

Sociological Abstracts:

((Ti,ab,su(LGBT* or Lesbian* or WSW or "Women having sex with women" or Gay or Gays or MSM or "Men having sex with men" or Homosexual* or Same-sex* or Bisexual* or MSMW or "Men having sex with men and women" or WSWM or "Women having sex with women and men" or Trans or Transgender* or Transsexual* or Transidentit* or Intersex* or Queer* or Genderqueer* or Non-binar* or "Gender fluid*" or "Sexual minorit" or "Gender minorit*" or SOGI* or "Sexual orientation*" or "Gender Identit*" or "Gender expression*" or "Sexual characteristic*") or MAINSUBJECT.EXACT("Sex Roles") OR MAINSUBJECT.EXACT("Sexuality") OR MAINSUBJECT.EXACT.EXPLODE("Bisexuality") OR MAINSUBJECT.EXACT.EXPLODE("Femininity") OR MAINSUBJECT.EXACT.EXPLODE("Homosexual Relationships") OR MAINSUBJECT.EXACT.EXPLODE("Homosexuality") OR MAINSUBJECT.EXACT.EXPLODE("Intersexuality") OR MAINSUBJECT.EXACT.EXPLODE("Lesbianism") OR MAINSUBJECT.EXACT.EXPLODE("Masculinity") OR MAINSUBJECT.EXACT.EXPLODE("Sex Differences") OR MAINSUBJECT.EXACT.EXPLODE("Sex Role Attitudes") OR MAINSUBJECT.EXACT.EXPLODE("Transsexuality")) AND (((MAINSUBJECT.EXACT("Courts") OR MAINSUBJECT.EXACT("Law") OR MAINSUBJECT.EXACT("Norms") OR MAINSUBJECT.EXACT("Policy") OR MAINSUBJECT.EXACT("Rights") OR MAINSUBJECT.EXACT("Treaties") OR MAINSUBJECT.EXACT("Human Rights") OR MAINSUBJECT.EXACT("International Law") OR MAINSUBJECT.EXACT("Legislation") ) and (

MAINSUBJECT.EXACT("Developing Countries") OR MAINSUBJECT.EXACT("European Union") OR MAINSUBJECT.EXACT("International Cooperation") OR MAINSUBJECT.EXACT("International Law") OR MAINSUBJECT.EXACT("International Organizations") OR MAINSUBJECT.EXACT("Internationalism") OR MAINSUBJECT.EXACT.EXPLODE("Cultural Groups") )) or ( noft(Convention* or Court or Courts or Law or Laws or Legislat* or Norm* or Policy or Policies or Recommendation* or Resolution* or Right* or Treaties or Treaty) near/5 noft(America* or Carribbean* or Europe* or Africa* or Asia* or Arab* or Oceania* or "United Nations" or UN or "U.N." or Global* or International or Regional ) ))) NOT (Ti,ab,su("review essay*" or "book review*" or “editorial*” or “letter*”))

PAIS:

(Ti,ab,su(LGBT* or Lesbian* or WSW or "Women having sex with women" or Gay or Gays or MSM or "Men having sex with men" or Homosexual* or Same-sex* or Bisexual* or MSMW or "Men having sex with men and women" or WSWM or "Women having sex with women and men" or Trans or Transgender* or Transsexual* or Transidentit* or Intersex* or Queer* or Genderqueer* or Non-binar* or "Gender fluid*" or "Sexual minorit" or "Gender minorit*" or SOGI* or "Sexual orientation*" or "Gender Identit*" or "Gender expression*" or "Sexual characteristic*" ) or MAINSUBJECT.EXACT("Intersexuality") OR MAINSUBJECT.EXACT("Sexuality") OR MAINSUBJECT.EXACT.EXPLODE("Bisexuality") OR MAINSUBJECT.EXACT.EXPLODE("Femininity") OR MAINSUBJECT.EXACT.EXPLODE("Homosexual Relationships") OR MAINSUBJECT.EXACT.EXPLODE("Homosexuality") OR MAINSUBJECT.EXACT.EXPLODE("Lesbianism") OR MAINSUBJECT.EXACT.EXPLODE("Masculinity") OR MAINSUBJECT.EXACT.EXPLODE("Sex Differences") OR MAINSUBJECT.EXACT.EXPLODE("Sex Role Attitudes") OR MAINSUBJECT.EXACT.EXPLODE("Sex Roles") OR MAINSUBJECT.EXACT.EXPLODE("Sexual Minorities") OR MAINSUBJECT.EXACT.EXPLODE("Transsexuality")) AND (((MAINSUBJECT.EXACT("Courts") OR MAINSUBJECT.EXACT("Law") OR MAINSUBJECT.EXACT("Policy") OR MAINSUBJECT.EXACT("Rights") OR MAINSUBJECT.EXACT("Treaties") OR MAINSUBJECT.EXACT("Human Rights") OR MAINSUBJECT.EXACT("International Court") OR MAINSUBJECT.EXACT("International Law") OR MAINSUBJECT.EXACT("Norms") ) and ( MAINSUBJECT.EXACT("Intergovernmental Relations") OR MAINSUBJECT.EXACT("International Economic Organizations") OR MAINSUBJECT.EXACT("International Organizations") OR MAINSUBJECT.EXACT.EXPLODE("Commonwealth of Independent States") OR MAINSUBJECT.EXACT.EXPLODE("Cultural Groups") OR MAINSUBJECT.EXACT.EXPLODE("International Cooperation") OR MAINSUBJECT.EXACT.EXPLODE("International Courts") OR MAINSUBJECT.EXACT.EXPLODE("International Law") OR MAINSUBJECT.EXACT.EXPLODE("Internationalism") )) or ( noft(Convention* or Court or Courts or Law or Laws or Legislat* or Norm* or Policy or Policies or Recommendation* or Resolution* or Right* or Treaties or Treaty ) near/7 noft(America* or Carribbean* or Europe* or Africa* or Asia* or Arab* or Oceania* or "United Nations" or UN or "U.N." or Global* or International or Regional or Commission* or Council*) ))

Web of Science:

TS=(LGBT* or Lesbian* or WSW or "Women having sex with women" or Gay or Gays or MSM or "Men having sex with men" or Homosexual* or Same-sex* or Bisexual* or MSMW or "Men having sex with men and women" or WSWM or "Women having sex with women and men" or Trans or Transgender* or Transsexual* or Transidentit* or Intersex* or Queer* or Genderqueer* or Non-binar* or "Gender fluid*" or "Sexual minorit" or "Gender minorit*" or SOGI* or "Sexual orientation*" or "Gender Identit*" or "Gender expression*" or "Sexual characteristic*") AND((Convention* or Court or Courts or Law or Laws or Legislat* or Norm* or Policy or Policies or Recommendation* or Resolution* or Right* or Treaties or Treaty) NEAR/7 (America* or Carribbean* or Europe* or Africa* or Asia* or Arab* or Oceania* or "United Nations" or UN or "U.N." or Global* or International or Regional or Commission* or Council* ))

## Appendix: Detailed documents

| Authors | Institutions, conventions, others | Analysis | Main results |
| --- | --- | --- | --- |
| (Abrusci 2017) | ICCPR | Case-law review: Nicholas Toonen, Edward Young, X | - Nicholas Toonen v. Australia as the basis of HRC’s condemnation of discrimination based on sexual orientation - Convergence of the case-law of international and regional courts around sexual orientation - Risks of fragmentation of case-law between international and regional courts with increasing complaints |
| (Baisley 2016) | UNGA, UNHRC | Review of policies pertaining to SOGIE within UN | - Emerging norms: human rights principles of universality, non-discrimination and equality including SOGI issues + prioritisation around death penalties + prohibition against violence and discrimination + obligation to respond to these violations - Norms constructed by states = less radical but more effective than those made by UN experts + CSO - UN experts = not as progressive + CSO = not as effective - Use of SOGIESC language as a bargaining tool (to divide women from lesbian for e.g.) |
| (Banda et Eekelaar 2017) | UDHR, ICCPR, ICESCR, CEDAW | Case study: Family in international laws | - Importance of anti-discriminatory norms in the parent-child relationship in distinguishing “legitimate” and “illegitimate” families - Extension of non-discriminatory principles from unmarried heterosexual couples to same-sex couples |
| (Bassetti 2020) | ICCPR, UNHCR | Queering international laws in relation to CIDT | - Distinction between Torture, Cruel and Inhuman or degrading treatment deepens on the nature, purpose and severity of the treatment - Pathologisation, and imposition of non-consensual medical interventions as requirement for legal gender recognition falls within the prohibition of TCIDT - Prohibition to obtain Legal gender recognition = cruel and inhuman or degrading treatment via the violation of the right to privacy and by being compelled to conceal one’s gender identity (resulting in psychological harm). - Coercive sterilisation presents double standards for women (right to be free from CIDT) and trans* (right to private life). |
| (Bauer, Truffer, et Crocetti 2020) | CRC, CAT, CEDAW, OHCHR, Yogyakarta | Case study: Intersex genital mutilation at the UN | - Categories having the most resonance for intersex rights: harmful practices, prohibition of TCIDT, integrity of the person, access to justice, involuntary medical experimentation, violence against children, right to life, coercive sterilisation, right to health, rights of the child, anti-discrimination laws that includes sex characteristics - Difficulties in implementing intersex rights comes from the need to depathologise intersexuality. |
| (Bejzyk 2017) | UNGA, UNHRC, OHCHR, CAT | Case study: TCID and criminalising laws | - Criminalising laws are often viewed as a violation of states’ obligation to protect the right of privacy - Criminalising laws are often viewed as a violation of the guarantee of equality and non-discrimination - Increased number of cases about the right to freedom from TCID - Criminal laws foster stigmatisation and increase vulnerabilities - Need to add TCID arguments to the “privacy, equality, non-discrimination” paradigm |
| (Biddolph 2024a) | ICC | Queering the ICC | - Recognition of anti-queer violence outside and within transitional justice - Importance of LGBTQIA+ people in participating in transitional justice - Need to queer and decolonise transitional justice (underlined cis-heteronormative, colonial and carceral violence) |
| (Biddolph 2024b) | ICC (for the former Yugoslavia) | Queering the International Criminal Tribunal for the former Yugoslavia | - Queer lives are present in the International Criminal Tribunal for former Yugoslavia, via their absence - There is a need to address this absence through deconstructing cis-heteronormativity |
| (Boulos et González-Cantón 2022) | CAT, CRC, UNHRC, CESCR, UPR | Queering the concepts of TCIDT | - Must prohibit sexual orientation change efforts (SOCE), as these practices relate to TCIDT - States are obliged under various treaties to ban any form of TCIDT - Definition of TCIDT can be applied to SOCE (whether harder/softer practices). - Consent of SOCE cannot serve as a justification since prohibition of torture is peremptory and the states’ obligations to eradicate discriminatory laws and practices against LGBT populations. |
| (Calazans 2022) | WHO, UNAIDS | Historical review of the use of MSM | - The use of MSM in the field of HIV/AIDS was motivated by an epidemiological approach and programmatic objectives - The use of MSM homogenised and invibilised gay, bisexual et trans* communities, erasing specific identities, political fights and realities |
| (Carpenter 2016) | CRC, CAT, CRPD, WHO | Comment: rhetoric and practices around intersexuality | - Importance to develop human rights-based medical pathways - Arguments are around bodily autonomy, stigmatisation and medicalisation |
| (Carpenter 2018) | WHO (ICD), UPR | Review of ICD | - “Disorders of sex development” was thought to be pejorative and inappropriate, leading to negative effects on access (avoidance) to healthcare and research - Need of an umbrella term for intersex realities to be more neutral (to better access healthcare), facilitate social-support and collective actions, and outside medical sphere - Nomenclature can lead to normalisation surgeries, which are unnecessary, often irreversible, and not consented for. - Need for new code on “intersex genital mutilation” |
| (Carpenter 2020) | Yogyakarta | Queering (intersex) Yogyakarta | - SOGIE rights failed to protect intersex rights (which can be more specific to harmful practices in medical settings) |
| (Carvalho 2024) | ICCPR, Yogyakarta | "Draging" international law-making | - Yogyakarta Principles are tools to challenge international law and establish a form of custom, a prerequisite to the production of international benchmarks for future jurisprudence - Imitation and repetitiveness serves cis-heteronormativity, which is challenged by the Yogyakarta Principles |
| (Castro-Peraza et al. 2019) | WHO (ICD-DSM), Yogyakarta | Comment: Pathologisation of trans* | - Pathologisation of SOGIESC infringes human rights (civil, economic, socio-cultural and the access to health care) - Discrimination can be linked to pathologizing diagnostic classifications - Poor access to trans-health is stigma associated with mental illness (need of legal gender via psychiatric diagnosis) - Pathologisation is unfounded due to the needlessness of treatment (gender incongruence-dysphoria) |
| (Chen 2024) | IE SOGI, UNHCR | Case study of a roundtable as a site for normalisation | - The roundtable was a site for norm emergence, for multiple sectors (humanitarian and human rights) |
| (Chernova 2024) | Geneva Conventions | Queering international humanitarian law (Geneva conventions) | - Neutrality and impartiality as inhibitors to radical change in gender norms and power relations - Importance to deconstruct the role of women and LGBITQ+ populations in relation to the Geneva Conventions |
| (DeLaet 2019) | UDHR, Yogyakarta | Case study: Soft laws | - Soft laws are critical to gender-based human rights due to socio-cultural inequities and patriarchy - Localised politics drive productive change over absent authoritative laws - Importance of socio-cultural transformation and norm diffusion - LGBTQ hard laws are absent due to the insufficient support among many countries in UN |
| (Duffy 2021) | CEDAW, Yogyakarta, ICCPR, HRC, UNGA, OHCHR, IE SOGI, CESCR, UPR | Linguistic review of UN policies | - Significant risk of invisibilising GI when talking about SOGI or LGBT. - Importance of treaty bodies and special procedures (IE SOGI) in representing SOGIESC issues - Importance of UPR in discussing SOGIESC - CEDAW challenges male/female hierarchies remaining in a binary conception of gender - Construction of gender-variant people as “victims of discrimination”, through “pathologisation” and “westernisation” |
| (Đuric, Vidlička, et Bogush 2018) | ICCPR, ICESCR, ICC Statute | Queering the ICC Statute | - Legal qualification of persecution on grounds of sexual orientation and gender identity is in line of today’s international human rights frameworks. |
| (Endsjø 2020) | HRC | Review of LGBT rights vs freedom of religion | - Religions are not entirely against LGBT rights and provide a diversity of traditions. Pro-LGBT beliefs are part of religions (positive protection) - There is a freedom “not” to be religious and practice religion (negative protection) - Need to change the paradigm of Religion vs LGBT (how freedom of religion can protect LGBT rights) - Discriminatory practices infringe on the freedom of religion for LGBT people |
| (Gerber et Timoshanko 2021) | CRC | Review of concluding observations and general comments | - Three groups of rights under the CRC (participation, provision and protection) - 36,5% of Concluding observations of the CRC mention LGBT issues (vs 33,8% for the HRC) - The intersection for CRC and LGBT issues are: Policies and strategies to combat discrimination, law reform, access to healthcare, awareness raising and education, and combatting violence and abuse. - Need systematic approach to the rights of LGBT children and children with LGBT parents - Need specific recognition of LGBT issues (specific general comment, section in concluding observations reports) |
| (Gillett-Swan et van Leent 2019) | CRC, UNICEF | Queering CRC | - Best interest, participation, non-discrimination and right to life, survival and development are intersecting with heteronormativity and childhood innocence - Lack of information for early childhood and comprehensive sexuality - Education is essential in contribution to normalise diverse representations of gender and sexuality |
| (Hagen & O'Rourke 2023) | ICCPR, CEDAW, ICESCR, Yogyakarta, UNSC | Queer critique of the Women, Peace and Security agenda at the UNSC | - The WPS illustrates clear examples of queer exclusions in international law, the gender binary, heteronormativity and invisibility of lesbian and non-normative gender people - The focus on sexual "violence" emphases "sexual danger", and is a base for queer exclusion in international law |
| (Heinze 2022) | UDHR, ICCPR, Yogyakarta, HRC | International human rights interpretation under libertarianism | - International human rights law is infused by both libertarianism (individual human rights) and human rights (understood as social welfare) - LGBTQ+ rights can fall under a libertarian point of view (defending individual liberties over "public morality") |
| (Joosten 2024) | CRC | Queering the UNCRC | - Protectionist and heteronormativity in the UNCRC limit the expression of queer children and their access to justice - Queer children's experiences are disregarded by a binary lens, a social construct of childhood being asexual, immature, innocent, and symbol of the future |
| (Kirichenko & Krol 2022) | CRPD | Intersectional queering of the CRPD | - There is a greater quantity of discourses in the CRPD towards SOGIESC inclusion - The discourses at the CRPD have changed to become more aware of inequalities lived by SOGIESC diverse populations, but remains limited to certain aspects (right to non-discrimination and integrity of the person) |
| (Kirichenko 2023) | CEDAW, CERD, CRPD, | Intersectional queering of the CEDAW, CRPD, CERD | - CEDAW, CRPD, and CERD are treaty bodies less in contact with SOGIESC issues, but are interesting sites to mobilise due to their respective specificities: focus on national bias and gender stereotypes, importance of participatory approaches, and potential site to valorise non-Western SOGIESC |
| (Leddy 2022) | Geneva conventions, ICC | Multiple case study of the ICC and SOGIESC | - Gender persecution in the Rome Statute includes sexual orientation and gender identity (as social construction of gender) - While having a binary lens of sex and gender, the ICC can include SOGIE based discrimination grounds - See ten recommendations for legal investigation, charging and trial of SOGIESC cases |
| (Lee 2022) | ICCPR, Yogyakarta, UNHCR, WHO | Queering the international laws through a "minority" lens | - Minority stress can be correlated to health inequities, through minority-dominant values and conflict - Absence of communities not having sexualities or genders in the international laws - Need to instore "Sexual and Gender Minorities" as a legal concept to represent absent communities |
| (Lee 2023) | WHO | Discourse analyses: sexual and gender minorities and the sociology of ignorance in the WHO | - “Not knowing” as a strategy to avoid acting towards LGBT rights - Commissive mis-recognition = when SGM members are purposefully uncounted (intentionally doing nothing) - Omissive non-recognition = when SGM members’ existence is unintelligible, therefore uncountable. (unintentionally non-doing anything) - Mis-recognition = we know but do not consider - Non-recognition = we do not know - “Absence of evidence” is a good indicator for epistemic exclusion |
| (Linde 2019) | CRC | Queering CRC’s notion of sexual agency | - Queer children do not have sexual agency because their identity and expression is sexualised (regulated through cultural boundaries) and are excluded from adult identities |
| (Lyons & Christiancy 2022) | Yogyakarta, UNHRC, IE SOGI | Case study of the Standards of conduct for Business: Tackling discrimination against LGBTI people | - The Standards should be used as human rights tools to foster LGBTI equality - Need to expand our consideration of SOGIESC in business through further participation of concerned and most marginalised populations |
| (Macdonald et al. 2022) | WHO | Review of WHO's commitments to trans* and gender diverse peoples' health | - WHO's understanding about trans* communities' access to universal health coverage has evolved and can be further improved through trans* communities' involvement |
| (Margalit 2018) | Geneva conventions, UNHRC,  UNSC | Queering international laws on armed conflict | - LGBT people are protected under international humanitarian laws, and parties are obligated to provide human treatment to them. - States must be reminded of their obligations through the support of notions such as humanity, inherent dignity of every human being, non-discrimination and non-violence. |
| (McGoldrick 2016) | UNGA, HRC, UPR, ICCPR, Yogyakarta | Case study: development of SOGIE issues in IHRL | - Key strategies for bridge polarised views on SOGIESC:   - - Focus on uniting states (over dividing them)     - Promotion of dialogue over confrontation     - Highlight economic issues     - More reports and awareness within UN organisations     - Focus on domestic jurisprudence for greater legitimacy and authority     - Promote CSOs     - Transform public attitudes |
| (Mulé, McKenzie, et Khan 2016) | ECOSOC, OHCHR, UNHCR, UN women, UNHRC | Case study of UN policies through advocacy, bureaucracies and member states’ role | - Recognition of LGBTIQ+ issues is made mainly in ECOSOC and OHCHR - Legitimation of these issues is hindered by unaware UN bureaucracy and member states’ opposition - Homonationalism hinders advocacy in other contexts than global north |
| (Nugraha 2017) | ICCPR, CRC | Queering CRC regarding SOCE | - SOCE’s detrimental effects are scientifically proven and should account in the determination of a child’s best interest - Need to better link SOCE to TCIDT (especially psychological pain) |
| (O'Connor et al. 2022) | CEDAW, Yogyakarta, UNHRC | Review of trans*, gender binary in international law | - Trans* communities need active, free and meaningful spaces to participate in their health improvement (this is at all stages of health research and interventions) - Need to apply a non-binary approach to international law to diminish trans* violence |
| (Paechter 2021) | CRC | Review of trans and intersex children’s rights and best interest | - Trans and intersex children present different realities:   - - Trans = not a natural act so “wait and see”, and use of policy to regulate the body - Regulations are made to prevent irreversible medical intervention, before the child has free and inform consent to proceed - The rights to participate in personal decisions are often ignored - Need higher focus on right to non-discrimination, identity, health, and to be heard (over the best interest of the child) |
| (Pauselli & Urzua 2024) | UNHRC, IE SOGI | Case study on autocracies and SOGIESC at the UNHRC | - The more a state is autocratic, the more likely it will oppose SOGI rights at the UNHRC. It is similar for democratic states with stronger far-right movements or social conservatism Autocracies form an important barrier to SOGI rights at the UNHRC level, favouring national sovereignty, social conservatism or new global order |
| (Pereira 2022) | ICCPR, CEDAW, CAT, CRC, CRPD | Case study on intersex activism within the UN | - Violences against intersex populations (e.g., medical procedures, killings) are widely recognised in UN treaty bodies but fail to address the binary system norming bodies. - Need to apply a non-binary approach to international law to diminish trans* violence |
| (Peretko 2024) | ICC | Queering international criminal law | - Need to integrate SOGIE in the notion of "gender" even if it deradicalize queer notions of SOGIE |
| (Pincock 2021) | UNHCR | Case study: UNHCR in the protection of LGBTIQ+ refugees in Kenya | - LGBTIQ+ sexuality can be homogenised to fit western notions - Need for a more emic perspective around LGBTI refugees lived experiences - UNHCR sustain a system of inequalities (heterosexism, racism) |
| (Ravesloot 2024) | UPR | Case study of UPR recommendations on intersexuality | - Need to further integrate the notion of "informed consent" in the recommendations against IGM to improve persuasiveness and level of acceptance - "Informed consent" must be genuine in that sense that it is informed (the family and community), in a competent manner (valorising participation in the decision) and voluntary |
| (Roos et Mackay 2016) | ICCPR | Review of same-sex marriage interpretations | - Conventions should be interpreted with an evolutionary interpretation (e.g., article 23 of ICCPR on marriage), based on good faith, in relation to its objectives and purposes (men and women interpreted as groups and not individuals), and according to the contexts (increased national recognition of non-heterosexual couples). - Unjustifiable arguments: marriage = procreation, need to protection traditional family and marriage, freedom of religion. |
| (Roos et Mackay 2019) | ICCPR | Case-law review: Juliet Joslin et al., C, G. | - Increased protection of the notion of privacy (individual and of a family) by the HRC (need to have proportionate and legitimate end to interfere with privacy) - Clearer recognition of same-sex marriage by establishing discrimination based on marital status (G. v. Australia) or international validity in foreign contexts (Fiona Campbell v. Australia). - Non usage of Juliet Joslin et al. v. New Zealand and article 23 (2) in decisions (C + G. v. Australia) |
| (Rossouw 2020) | Geneva conventions | Queering the Geneva conventions | - Identification of key concepts in IHL/IHRL: “respect”, “protection”, “humane treatment”. - Article 3: need for non-discrimination via “any other similar criteria” - Need to apply these concepts and article 3 towards the protection of LGBTIQ+ detainees |
| (Seckinelgin 2018) | OHCHR | Case study of same-sex lives in Africa with international lgbt rights | - UN reports and statements provide affirmative language towards LGBT rights that can create categories of states (non/respect of rights). - Foreign language and dialogue can disenfranchise LGBT communities abroad and put politicians as interlocutors - Paternalism is both from international norms (claiming international categories) and local political actors (claiming the foreignness of LGBT communities) |
| (Simm 2020) | CEDAW, ICCPR, CAT | Queering CEDAW through legal cases | - Incorporating queer issues to legal frameworks risks de-radicalising them. |
| (Singh et al. 2022) | Yogyakarta, CRC, CRPD | Review of intersex human rights via patient care | - International law affects patient care of intersex populations, therefore creating the need for medical practitioners to be aware of human rights-based practices |
| (Smith 2017) | HRC, UNGA 3^rd^ committee (social, cultural and humanitarian) | Case study: UN groups on SOGIE issues | - Three formal groups (OIC, Africa group, and EU) are leading the debates around “traditional values and human rights”, “protection of the family”, and “sexual orientation and gender identity”. - Both sides are coalescing with informal groups (Friends of the Family, LGBT core group) |
| (Sterling 2021) | UDHR, ICESCR | Literature review on intersexuality in education | - Need to invoke the right to education and to full development for intersex people’s education and safer spaces in the educational systems |
| (Stevens et Forrest 2018) | WHO, UNODC, UNAIDS, UNHRC | Review of chemsex responses in international policies | - Consensus-basis decision-making makes it difficult to refer to gbMSM in relation to drugs. - Progressive language can be employed to reduce “national contexts” mentions and to ask for repealing criminalising and stigmatising key populations |
| (Suess Schwend 2020) | WHO, Yogyakarta | Review on trans pathologisation | - Depathologisation is highly linked to human rights from a trans perspective, with priorities such as: right to bodily integrity and autonomy, and participation in health policies. - Trans depathologisation contribute to gender non-binarism, decolonisation, children’s human rights and legal gender recognition, clinical/research practices and language, etc. |
| (Voss 2018) | IE SOGI, UNHRC | Case study: SOGI a the UNHRC | - States advocate for/against SOGI issues by framing the issues through their “universal” perspective - Proponents legitimate SOGI issues through their adherence to prior international laws in a context of universal human rights. - Opponents delegitimate SOGI issues by arguing for relativist point of view, where the “West” is imposing its ideas. - Opponents are coalescing around the notion of “family” and its “traditional” definition - Populism in more progressive countries can hinders the advancement of SOGI resolutions |
| (Zelayandia-Gonzalez 2023) | ICCPR, CEDAW, ICESCR, CAT, CRC, CERD, CRPD | Review of intersexuality in concluding observations and recommendations | - There is an increased visibility of intersexuality in concluding observations made by treaty bodies - Visibility is mainly around the issues of IGM, autonomy and bodily integrity, and reparations around human rights violations |

## Appendix: Legal decisions from treaty bodies

| Years | Name | Treaty bodies | Articles brought forth (Decisions) | Claims made | Impacts |
| --- | --- | --- | --- | --- | --- |
| 1979-1982 | [Leo Hertzberg](https://juris.ohchr.org/casedetails/337/en-US) et al. v. Finland | HRC | 19  (no violation) | Interference with the right to hold opinions and freedom of expression | - First formally addressed case about sexual orientation at HRC - Radio programme is censured because it “encourages homosexual behaviour” - Dissenting opinion stating that “public morals” are subject to change and relative, and that minority views (even if offending, shocking, or disturbing to the majority) should be protected |
| 1991-1994 | [Nicholas Toonen](https://juris.ohchr.org/casedetails/702/en-US) v. Australia | HRC | 2 (1)  (Violation) | Discrimination based on other status | - Statements on:   - The interference with privacy of a law against private homosexual behaviour   - Criminalisation of homosexuality is not a reasonable means to prevent HIV/AIDS   - Moral issues are of concern for the HRC due to their connection to privacy   - Unenforced provisions against homosexual behaviours implies the absence of the need to protect morality.   - “Sex” status includes “sexual orientation” - Need to repeal offending law |
|  |  |  | 17 (1)  (Violation) | Violation of the right to family and privacy |  |
|  |  |  | 26  (Unnecessary to consider) | Inequality before the law because of discrimination based on other status : “sexual activity”, “sexual orientation”, and “sexual identity”. |  |
| 1998-2002 | [Juliet Joslin et al.](https://juris.ohchr.org/casedetails/995/en-US) v. New Zealand | HRC | 2 (1)  (No violation) | Discrimination based on sex | Individual opinion of 2 Committee members:   - Discrimination based on “sex” encompasses discrimination based on “sexual orientation” - Denial of certain rights or benefits to same-sex couples that are available to married couples may amount to discrimination |
|  |  |  | 16  (No violation) | Non-recognition as proper subjects of law, as persons and couples |  |
|  |  |  | 17  (No violation) | Violation of the right to family and privacy |  |
|  |  |  | 23 (1)  (No violation) | Lack of recognition and protection of (same sex) families |  |
|  |  |  | 23 (2)  (No violation) | Interpretation of “men and women” as groups |  |
|  |  |  | 26  (No violation) | Inequality before the law because of discrimination based on “sex” and indirectly “sexual orientation” |  |
| 1999-2003 | [Edward Young](https://juris.ohchr.org/casedetails/1076/en-US) v. Australia | HRC | 26  (Violation) | Inequality before the law because of discrimination based on “sexual orientation” | - Redefinition of “dependent”, including same-sex relationships - Recognition of “member of a couple” for same-sex partners |
| 2001-2007 | [X](https://juris.ohchr.org/casedetails/1338/en-US) v. Colombia | HRC | 2 (1)  (Unnecessary to consider) | Discrimination based on sex and other status | Recognition of discrimination in granting pension transfer in the case of homosexual couples |
|  |  |  | 3  (Not sufficiently substantiated) | Lack of enjoyment of all civil and political rights |  |
|  |  |  | 5 (1-2)  (Incompatible and inadmissible) | Failure to respect the principles of equality and  non-discrimination |  |
|  |  |  | 14 (1)  (Not sufficiently substantiated) | Violation of the right to equality before the courts |  |
|  |  |  | 17 (1-2)  (Unnecessary to consider) | Violation of the right to privacy |  |
|  |  |  | 26  (Violation) | Inequality before the law because of discrimination based on other status : sexual orientation |  |
| 2007-2011 | [Uttam Mondal](https://juris.ohchr.org/casedetails/52/en-US) v. Sweden | CAT | 3  (Violation) | No State shall extradite someone, somewhere he/she would be subjected to torture | Recognition that an expulsion to Bangladesh of a political activist homosexual man, part a of a Hindu minority, would amount to creating a risk for torture |
|  |  |  | 16  (not sufficiently substantiated) | State shall prevent CIDT |  |
| 2008-2011 | [X](https://juris.ohchr.org/casedetails/1390/en-US) v. Afghanistan, Sweden | HRC | 6  (Violation) | Violation of the right to life | - State needs to reassess the refoulement of a gay Afghan based on the risk of TCIDT - Focuses on inconsistencies and low credibility (late reveal of homosexuality) must be more appropriately weighted against allegations of risk of TCIDT |
|  |  |  | 7  (Violation) | Neglected assessment of persecution or torture and other cruel, inhuman or degrading treatment or punishment upon deportation |  |
| 2010-2012 | [Irina Fedotova](https://juris.ohchr.org/casedetails/1272/en-US) v. Russian Federation | HRC | 19 (3)  (Violation of article 19 (2)) | Violation of the right to freedom of expression | - Moral grounds must be derived from multiple social, philosophical and religious traditions, therefore, limitations to protect morality cannot be derived from a single tradition - No evidence that restriction on the right to freedom of expression in relation to “propaganda of homosexuality” among minors – as opposed to propaganda of heterosexuality or sexuality generally – is based on reasonable and objective criteria. |
|  |  |  | 26  (Violation) | Inequality before the law because of discrimination based on other status : sexual orientation |  |
| 2009-2013 | [Nikolai Alekseev](https://juris.ohchr.org/casedetails/1686/en-US) v. Russian Federation | HRC | 21  (Violation) | Violation of the right to peaceful assembly | Risk of a violent counterdemonstration (anti-LGBTIQ+) or the mere possibility that the authorities would be unable to prevent or neutralise such violence is not sufficient to ban a demonstration |
| 2012-2013 | [M. I.](https://juris.ohchr.org/casedetails/1675/en-US) v. Sweden | HRC | 7  (Violation) | Neglected assessment of persecution or torture and other cruel, inhuman or degrading treatment or punishment upon deportation | - Although national laws are not systematically applied, they reinforce the general climate for homophobia and impunity for LGBT persecutors. - Criminalising national laws shows the unwillingness and inability of the State to protect LGBT individuals - Reality of feared risks surpasses inconsistencies in the facts reported |
| 2013-2015 | [J.K](https://juris.ohchr.org/casedetails/2064/en-US). v. Canada | CAT | 3  (Violation) | No State shall extradite someone, somewhere he/she would be subjected to torture | - Recognition that returning a militant gay man to Uganda brings a risk of torture and ill-treatment during detention |
| 2010-2016 | [Sergei Androsenko](https://juris.ohchr.org/casedetails/2103/en-US) v. Belarus | HRC | 19  (Violation of article 19 (2)) | Interference with the freedom of expression | Recognition of a disproportionate and unnecessary apprehension limiting freedom to assembly and of expression |
|  |  |  | 21  (Violation) | Violation of the right to peaceful assembly |  |
| 2014-2016 | [M.K.H.](https://juris.ohchr.org/casedetails/2169/en-US) v. Denmark | HRC | 7  (Violation) | Neglected assessment of persecution or torture and other cruel, inhuman or degrading treatment or punishment upon deportation | State failed to account for a homosexual man’s version and the background context of Bangladesh in assessing risks of TCIDT in a non-refoulement case |
| 2011-2017 | [G](https://juris.ohchr.org/casedetails/2220/en-US). v. Australia | HRC | 2 (1;3)  (Unnecessary to consider 2 (1) and inadmissibility of 2 (3)) | Discrimination based on other status | - Privacy includes the right for gender identity - Denying married transgenders to change sex on the birth certificate is unreasonable and fails to provide equal protection - State needs to revise its legislation to ensure compliance with the Covenant |
|  |  |  | 17  (Violation) | Violation of the right to family and privacy |  |
|  |  |  | 26  (Violation) | Inequality before the law because of discrimination based on other status : marital status and transgender identity |  |
| 2012-2017 | [Fiona Campbell](https://juris.ohchr.org/casedetails/2375/en-US) v. Australia | HRC | 2 (1)  (Inadmissible) | Discrimination based on sex and other status | Recognition of foreign same-sex marriage as a recognised exception, equally to polygamous marriage |
|  |  |  | 14 (1)  (Unnecessary to consider) | Violation of the right to equality before the courts |  |
|  |  |  | 26  (Violation) | Inequality before the law because of discrimination based on other status : sexual orientation |  |
| 2013-2018 | [Kirill Nepomnyashchiy](https://juris.ohchr.org/casedetails/2546/en-US) v. Russian Federation | HRC | 19  (Violation) | Interference with the right to hold opinions and the freedom of expression | - The offence of “promoting propaganda of homosexuality” is too ambiguous to be lawful - To discriminate homosexuality from other sexual practices based on moral grounds is limited by the principle of non-discrimination |
|  |  |  | 26  (Violation) | Inequality before the law because of discrimination based on other status : sexual orientation |  |
| 2017-2020 | [O.N., D.P.](https://juris.ohchr.org/casedetails/2709/en-US) v. Russian Federation | CEDAW | 1  (Violation) | Discrimination based on sex and sexual orientation | - Obligations of States not only to take steps to eliminate direct and indirect discrimination, but to improve the *de facto* position of women - Discrimination against women is inextricably linked to other factors that affect their lives, including being lesbian - State party has a duty to modify or abolish not only existing laws and regulations, but also customs and practices that constitute discrimination against women, such as stereotypes |
|  |  |  | 2  (Violation) | Lack of appropriate legislative measures  Lack of legal protection  Lack of appropriate measures |  |
|  |  |  | 5  (Violation of 5 (a)) | Need to modify social and cultural that are based on sexism |  |
| 2018-2021 | [A.B](https://juris.ohchr.org/casedetails/2946/en-US). v. Finland | CRC | 2  (Not sufficiently substantiated) | Discrimination based on special characteristics  or other status of the child or his or her family | - State failed to properly assess the view and the best interest of a child, being the child of lesbian parents - Family threats and discrimination, and child bullying could amount to persecution |
|  |  |  | 3  (Violation) | Neglected assessment of the best interest of the child |  |
|  |  |  | 13  (Not sufficiently substantiated) | Lack of freedom of expression |  |
|  |  |  | 14  (Not sufficiently substantiated) | Lack of freedom of thought |  |
|  |  |  | 16  (Not sufficiently substantiated) | Lack of privacy |  |
|  |  |  | 17  (Not sufficiently substantiated) | Lack of access to obtain  information that is important to his health and wellbeing |  |
|  |  |  | 19  (Violation) | Lack of protection from being hurt  and mistreated, physically or mentally |  |
|  |  |  | 22  (Violation) | Lack of protection for a child seeking refugee status |  |
|  |  |  | 29  (Not sufficiently substantiated) | Lack of access to developing education |  |
| 2018-2022 | [Rosanna Flamer-Caldera](https://juris.ohchr.org/casedetails/3047/en-US) v. Sri Lanka | CEDAW | 2 (a;c-g)  (Violation) | Lack of appropriate legislative measures  Lack of legal protection  Lack of appropriate measures | - State must take steps to stop threats, harassment and abuse, by initiating criminal procedures against perpetrators targeting SOGIESC diverse women - State must ensure free and safe environment for SOGIESC activist organisation - State must decriminalise consensual same-sex sexual conduct between women passed the age of consent and protect diverse SOGIESC women from gender-based violence - State must provide training to law enforcement agencies on diverse SOGIESC gender-based violence |
|  |  |  | 5 (a)  (Violation) | Need to modify social and cultural that are based on sexism |  |
|  |  |  | 7 (c)  (Violation) | Failure to eliminate discrimination against women in the political and public life |  |
|  |  |  | 15  (Violation) | Failure to ensure that women have access  to the protection and remedies offered through criminal law, and that they are not exposed to discrimination within the context of those mechanisms |  |
|  |  |  | 16  (Violation) | Violation of the right to autonomy and choice |  |
